# Improving nanotherapy delivery and action through image-guided systems pharmacology

**DOI:** 10.7150/thno.37215

**Published:** 2020-01-01

**Authors:** Thomas S.C. Ng, Michelle A. Garlin, Ralph Weissleder, Miles A. Miller

**Affiliations:** 1 Center for Systems Biology, Massachusetts General Hospital, Boston, MA 02114.; 2 Department of Radiology, Brigham and Women's Hospital and Harvard Medical School, Boston, MA 02115; 3 Department of Radiology, Massachusetts General Hospital and Harvard Medical School, Boston, MA 02114.; 4 Department of Systems Biology, Harvard Medical School, Boston, MA 02115.

**Keywords:** Intravital microscopy, Magnetic resonance imaging (MRI), Positron emission tomography / computed tomography (PET/CT), Pharmacokinetics / pharmacodynamics, Tumor microenvironment, Enhanced permeability and retention effect (EPR effect), Nanomedicine

## Abstract

Despite recent advances in the translation of therapeutic nanoparticles (TNPs) into the clinic, the field continues to face challenges in predictably and selectively delivering nanomaterials for the treatment of solid cancers. The concept of enhanced permeability and retention (EPR) has been coined as a convenient but simplistic descriptor of high TNP accumulation in some tumors. However, in practice EPR represents a number of physiological variables rather than a single one (including dysfunctional vasculature, compromised lymphatics and recruited host cells, among other aspects of the tumor microenvironment) — each of which can be highly heterogenous within a given tumor, patient and across patients. Therefore, a clear need exists to dissect the specific biophysical factors underlying the EPR effect, to formulate better TNP designs, and to identify patients with high-EPR tumors who are likely to respond to TNP. The overall pharmacology of TNP is governed by an interconnected set of spatially defined and dynamic processes that benefit from a systems-level quantitative approach, and insights into the physiology have profited from the marriage between *in vivo* imaging and quantitative systems pharmacology (QSP) methodologies. In this article, we review recent developments pertinent to image-guided systems pharmacology of nanomedicines in oncology. We first discuss recent developments of quantitative imaging technologies that enable analysis of nanomaterial pharmacology at multiple spatiotemporal scales, and then examine reports that have adopted these imaging technologies to guide QSP approaches. In particular, we focus on studies that have integrated multi-scale imaging with computational modeling to derive insights about the EPR effect, as well as studies that have used modeling to guide the manipulation of the EPR effect and other aspects of the tumor microenvironment for improving TNP action. We anticipate that the synergistic combination of imaging with systems-level computational methods for effective clinical translation of TNPs will only grow in relevance as technologies increase in resolution, multiplexing capability, and in the ability to examine heterogeneous behaviors at the single-cell level.

## 1. Introduction

Therapeutic nanoparticles (TNPs) continue to successfully reach the clinic and impact the treatment of disease 1. Recent milestones include FDA approvals of the first TNP-encapsulated chemotherapy combination in 2017 (daunorubicin-cytarabine liposome, Vyxeos) for the treatment of acute myeloid leukemia, and the first RNA interference therapeutic in 2018 (patisiran, Onpattro), which utilizes a lipid nanoparticle (LNP) for hepatic delivery to treat polyneuropathy of hereditary transthyretin-mediated amyloidosis. Despite these and other notable successes, effective translation of TNPs for the treatment of solid cancers has been slow to accelerate since the clinical development of PEGylated liposomal doxorubicin in the 1990's 1. One increasingly appreciated contributing factor has been the heterogeneous and often inefficient delivery of nanomaterials into tumors. The promise of TNPs for the treatment of solid malignancies originally lay in their ability to extend systemic blood-half-lives, improve tumor targeting properties and avoid organ toxicities of small-molecule cytotoxic chemotherapies relative to their traditional solvent-formulated counterparts. In principle, nano-sized materials can enhance drug delivery into solid tumors through an amalgam of processes collectively referred to as the enhanced permeability and retention (EPR) effect. Drivers of the EPR effect include distortion and hyperpermeability of neovasculature, impaired lymphatic drainage, and local inflammation with recruitment of myeloid derived phagocytic cells, which together promote the ability of nanomaterials to durably accumulate in tumor tissue. Yet in practice, TNP delivery to tumors can be inefficient in many cases, and by some estimates there has been only modest improvement of the field's ability to predictably deliver TNPs into tumor tissue over the past decade 2, with the benefits for TNP delivery offered by EPR offset by other properties of the tumor microenvironment (TME), such as elevated interstitial fluid pressure (IFP) or perfusion impairment of the tumor vasculature. Such low or mixed tumor uptake of TNPs presents a major hurdle to clinical translation and highlights the need to develop a more fundamental and quantitative understanding of the complex interactions at play between NPs, the tumor, and other tissues in order to predict and optimize TNP action.

Imaging has played a vital role in understanding the delivery and action of nanomedicines 3-6, and preclinical nanomedicine studies often incorporate a component of imaging to quantify TNP biodistribution. Increasingly powerful high-resolution and multiplexed imaging techniques allow the dynamic processes of NP delivery, tumor extravasation, and interactions with the TME to be measured. However, direct comparison of findings across preclinical studies are limited by disparities in the types of nanomaterials studied, the variability across tumor models 7 along with the scope and scale of the parameters measured in any individual study 2. Extrapolation of preclinical findings to the clinic (and vice versa) is additionally impeded by our current limited ability to perform intravital microscopy (IVM) in patients, and because clinical imaging techniques lack cellular resolution at the level of IVM. Furthermore, many individual processes of *in vivo* TNP action are inter-connected and depend on one another. Thus, quantitative modeling frameworks offer a useful avenue for integrating results across studies and for interpreting how multiple aspects of TNP pharmacology integrate to influence their overall behavior.

Computational systems-level modeling of drug pharmacology is now a frequent component of clinical translation of any therapeutic drug and has even made its way into FDA regulatory decision making 8. Such approaches can be labeled as computational pharmacology, pharmacometrics, physiology-based pharmacokinetic (PBPK) modeling, and quantitative systems pharmacology (QSP), and these overlapping terms each carry historical associations and definitions 9. For simplicity, we use QSP very generally here to describe the broad range of mathematical modeling techniques to understand how drugs transport and behave across tissues and towards their targets. QSP insights at multiple levels have improved our understanding of the physiological processes governing the delivery of NPs to the tumor site, uptake of NPs via the EPR (and other biophysical processes) to the target cells of interest, and ultimately the action of the drug payload on its targets 10-14. If appropriate pharmacological models are developed, prediction and optimization of NP uptake can be aided with in silico computational simulations 14, thus streamlining the NP development process and guiding strategic laboratory and clinical studies. In this light, acquisition of robust *in vivo* imaging data takes on an added potential benefit by providing tangible data to populate and optimize these models 15.

In this article, we review recent developments pertinent to the field of image-guided systems pharmacology as applied to the study of nanomedicines in oncology. We first highlight recent developments in quantitative imaging technologies that enable pharmacokinetic and pharmacodynamic analysis of nanomaterials at multiple spatiotemporal scales. We then review recent studies that have adopted imaged-guided QSP approaches, in particular those that have integrated the use of multi-scale imaging with modeling to derive insights about the EPR effect, and studies that have used modeling to guide and understand the manipulation of the EPR effect as well as other systemic and TME properties for NP pharmacological enhancement. Image-guided QSP approaches used in recent NP clinical translational studies are examined. Finally, we discuss key challenges that need to be addressed in order to maximize the potential of an image-guided systems pharmacology approach to guide successful translation of nanotherapies for clinical use.

## 2. Quantitative imaging technologies

**Figure 1:**
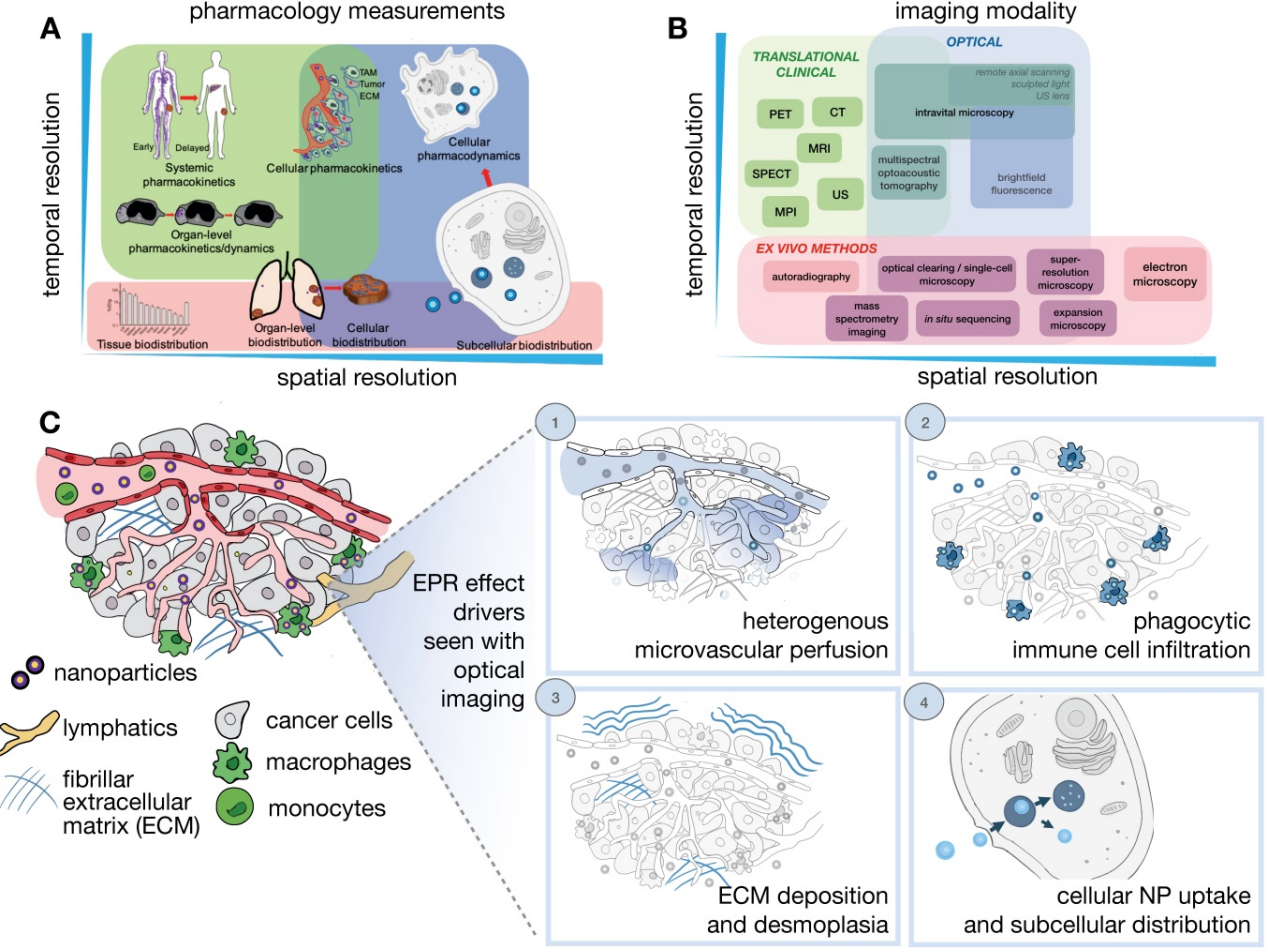
** Imaging NP pharmacology and the EPR effect across multiple spatial and temporal scales.** (**A-B**) Comprehension of the pharmacokinetics and pharmacodynamics (PK/PD) of NPs requires an understanding of physiological processes occurring at different dynamics and spatial length scales (**A**), therefore benefiting from the integrated application of multiple imaging modalities that capture these processes across a range of spatial and temporal resolutions (**B**). Imaging strategies include modalities appropriate for translational/clinical applications (green), as well as optical imaging and especially intravital microscopy (IVM), which allow single cell and subcellular processes to be visualized *in vivo* (blue). *Ex vivo* methods can complement *in vivo* assays by supporting improved resolution, tissue penetration, and multiplexed molecular labeling (red). (**C**) IVM techniques are especially suited for visualizing dynamic and microscopic processes of the EPR effect and the TME. This is highlighted in four examples, including: (1) Understanding the role of heterogeneous tumor vasculature in impacting NP extravasation at the tumor site, (2) Understanding the immune cell make-up of the TME 198, for example phagocytic cells such as tumor-associated macrophages, and their impact on NP penetration and drug release to surrounding cancer cells, (3) Understanding the impact of the extra-cellular tumor matrix in affecting NP penetration and clearance, and (4) Understanding the mechanisms of NP uptake in the target cells of interest.

Determinants of the EPR effect and TNP drug action play out across multiple spatial and temporal scales, ranging from systemic biodistribution of TNPs to their uptake and effects upon individual cells (**Figure 1A**). Unfortunately, no single imaging technique can fully accommodate the different levels of assessment necessary to comprehensively understand all aspects of NP pharmacology, and thus distinct imaging modalities spanning the assessment requirements across these scales are often combined to provide a complimentary and more complete perspective (**Figure 1B**). In general, *in vivo* imaging strategies require a compromise between image penetration depth, spatial and temporal resolution, and the possible types of image contrast. For clinical studies, patient tolerance for a particular protocol needs also to be considered. Here, we mainly focus on quantitative *in vivo* imaging technologies defined as those that either give cellular detail or those where the imaging signal is not significantly degraded by depth and scattering and can be used for whole body imaging.

Specifically, these techniques include laboratory technologies primarily based on confocal laser scanning microscopy (CLSM), as well as clinically relevant imaging modalities including positron emission tomography (PET) and magnetic resonance imaging (MRI). We recognize that several new exciting imaging techniques (e.g. light sheet microscopy, adaptive optical fluorescence microscopy, remote axial scanning, use of ultrasound lenses, optoacoustic lens imaging and magnetic particle imaging) are being developed that will likely extend the possibilities for imaging nanomaterials, and we refer readers to other recent reviews for further information 4, 15-17.

### 2.1. *In vivo* confocal (intravital) microscopy

IVM has been established as a collection of techniques (e.g. CLSM, multiphoton microscopy, epifluorescence) to dynamically assess tumor anatomy and physiological processes from a tissue- to subcellular-level perspective 4, 16 (**Figure 1C**). The availability of IVM setups enable simultaneous imaging of multiple TME components in conjunction with nanomaterial distribution, and we note several excellent in-depth reviews that cover this topic 18-20. With specific regards to understanding the EPR effect, several technical factors need to be considered. High resolution imaging of the TME, especially at the single cell level, provides vital insights of NP/TME interactions, but is often affected by animal motion. Strategies have been applied to mitigate cardiopulmonary motion in IVM 21, including physical stabilization/suction devices, respiratory/cardiac gating 22, real-time/post-hoc motion compensation algorithms 23, and immobilizing surgical windows and supports 21, which collectively enable prolonged imaging at subcellular resolution.

**Figure 2:**
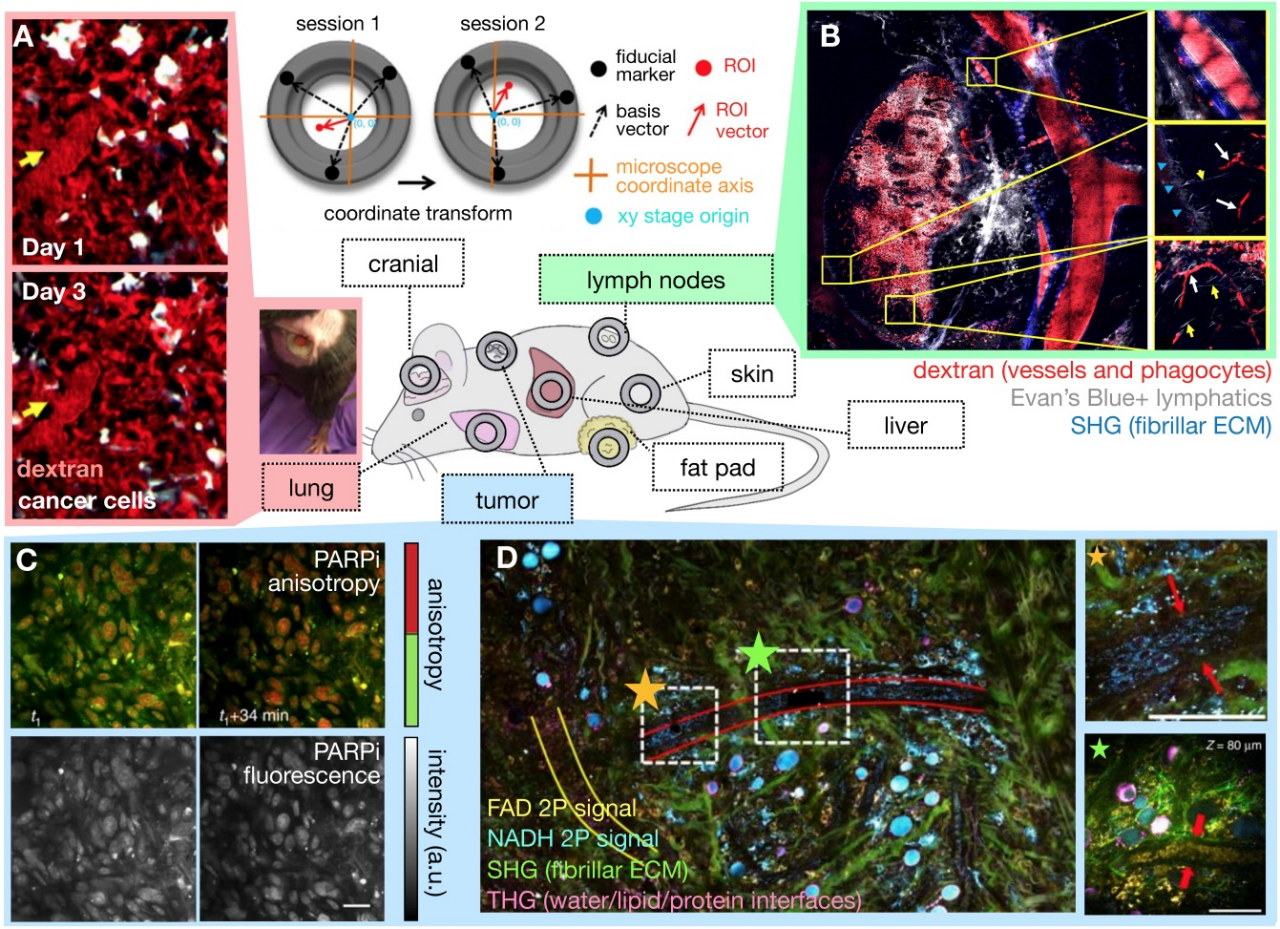
** Intravital microscopy (IVM) developments aid EPR assessment.** The availability of surgically implanted imaging windows at various anatomical sites allows tissue stabilization and longitudinal IVM of orthotopic disease sites over days and weeks (center). (**A**) Microcartography performed using fiducial marks etched on imaging windows enables precise localization across imaging sessions. Here, lung tumor vasculature was followed over multiple days. Yellow arrows indicate the location of the same microvessel branch point each day. A photograph of a long-term lung window is shown in a freely moving mouse (Adapted with permission from 26, copyright 2018 Springer Nature). (**B**) IVM mosaicking combines large scale and zoomed-in views of the TME. Here, a 10 x 10 mosaic covers a 4 x 4 mm lymph node area. Subcapsular sinuses are magnified at right, and shadows of erythrocytes (white arrows) and lymphatic capillaries (yellow arrows) are visible (Adapted with permission from 27, copyright 2017 Elsevier). (**C**) Real-time target engagement of the PARP inhibitor olaparib (PARPi) can be visualized *in vivo* using anisotropy imaging. High anisotropy (red) indicates the fluorescently-labeled PARPi has bound to a protein, which is localized in the nuclei of cancer cells (Adapted with permission from 199, copyright 2014 Springer Nature). (**D**) Multiphoton label-free IVM of a large tumor field (1.5 × 1.5 mm^2^) highlights cellular and ECM structures simultaneously (Adapted with permission from 40, copyright 2018 Springer Nature).

Although subcutaneous implantation facilitates tumor imaging and treatment in preclinical studies, it may not recapitulate the TME and hence EPR physiology present at orthotopic and metastatic sites. Tumor location context is increasingly recognized to affect TME constitution, including vascularity and immune cell composition. To address these issues, optical imaging approaches to access orthotopic disease sites are actively being developed. Interrogation of metastatic and orthotopic locations has been performed by surgical organ externalization, including of the pancreas 24 and the intestine 25, but many of these studies are terminal and only useful for monitoring through short time periods. For longitudinal imaging over days and weeks, window chambers have been developed in the cranium, chest and abdominal walls, over the mammary glands and in the dorsal skin 21 (**Figure 2**). These are especially useful for nanomaterial evaluation given their extended pharmacokinetics, prolonged drug payload release rates, and longer-term downstream tumor effects. One recent advance has been the development of a permanent thoracic window for long-term (days to weeks) imaging of lung metastases by Entenberg et al. 26 (**Figure 2A**). In this work, microcartography techniques were adapted to enable reliable localization of microvasculature between imaging sessions, and multiple stages of metastatic progression were visualized in a murine breast cancer model. To evaluate both bulk whole tumor and single cell features, tissue stabilization approaches and post-acquisition image processing using mosaic stitching techniques were developed that enabled large volume IVM covering a region of up to 4 x 4 mm, albeit with time resolution on the order of minutes 27 (**Figure 2B**). This approach represents an advance over suboptimal IVM setups that can only focus on a small tumor region, and therefore fail to capture the full extent of intratumoral TME features and heterogeneous NP behavior across a tumor. Imaging windows can require significant design and surgical expertise to setup and maintain. Nonetheless, such techniques can potentially offer insights into the impact of intratumoral EPR and TME heterogeneity on NP uptake.

Longitudinal high-resolution imaging enabled by IVM comes with risks for artifact that should be experimentally minimized, and caveats that should be considered during data interpretation. Surgical manipulations have the potential of creating inflammation, infection, and structural tissue damage 16. Window chambers may artificially mechanically compress tumor vasculature and lymphatics and distort NP delivery 21. Commonly used anesthesias for IVM include ketamine/xylazine and inhaled isoflorane, which can impact tumor vasculature and pressures. For example, isoflourane has been shown to attenuate permeability measurements using dynamic contrast-enhanced MRI (DCE-MRI) and diffuse reflectance spectroscopy measurements 28, whereas ketamine and propofol were shown not to affect intestinal microcirculation 29. Overall, these risks can be mitigated using careful and sterile surgical and anesthetic techniques, and key IVM findings are often supported by complementary models and experiments that do not rely on imaging or accompanying surgical manipulations 4, 5, 25, 30-32.

### 2.1.1. Imaging agents, methods and biological systems for intravital microscopy

The multiphoton and multichannel capabilities of IVM systems can be harnessed for simultaneous assessment of multiple NP and TME components 4, 5, 16, 32, 33. Nanomaterials can be fluorescently labeled for kinetic tracking 5, 30, 34, and combined with alternatively fluorescently labeled drug payloads to differentiate between the kinetics of the nano carrier and their encapsulated therapeutic 31, 35, 36. Care should be taken to ensure that the effect of labelling on the pharmacological behavior of NP or drug activity is minimized and well characterized. Fluorescence resonance energy transfer (FRET) 37, quenching 36, and anisotropy 38 effects between different fluorophores can distinguish NP-associated drugs from those which have released from their NP vehicle. For instance, fluorescently tagged small molecule drugs can exhibit increased fluorescence anisotropy upon binding to their protein targets, as a consequence of their higher apparent molecular weight and slower molecular rotation upon target complexation. This change in anisotropy can be visualized by IVM to detect drug binding at subcellular resolution (**Figure 2C**). Labeled nanomaterials can be combined with other exogenously introduced agents to characterize the TME, and fluorescent protein reporters of tumor cells (either introduced or bred into mice) 4, 5, other cellular subsets, as well as dynamic processes such as gene expression 39, protein dynamics or protein-protein interactions can be used 4, 5.

Label-free optical contrasts allow assessment of the TME using endogenous optical properties of tissue and cellular components. For instance, second harmonic generation (SHG) with multi-photon microscopy can visualize chiral structures such as fibrillar collagen, while coherent anti-Stokes Raman spectroscopy can visualize lipid 4. Recently, simultaneous label-free autofluorescence multi-harmonic (SLAM) microscopy was used to visualize endothelial cells and tumor vesicles along with SHG and third-harmonic generation (THG) signals 40. THG supports imaging of water-lipid and water-protein interfaces, while SHG elicits image contrast from non-centrosymmetric structures such as collagen. Using the SLAM technique, You et al. used two-photon autofluorescence to image nicotinamide adenine dinucleotide (NADH) and flavin adenine dinucleotide (FAD), SHG to image fibrillar ECM including collagen, and THG to image endothelial cells and leukocytes simultaneously from the same sample, enabling visualization of dynamic stromal and intercellular interactions (**Figure 2D**). Although the long wavelength laser typically used for THG is less often found in standard microscopy facilities, such approaches nonetheless open up possibilities of examining multiple facets of the TME along with the biodistribution of NP simultaneously.

### 2.2. Linking *in vivo* microscopy with *ex vivo* analysis

#### 2.2.1. Electron microscopy

Electron microscopy (EM) is extensively used to study nanomaterials. Transmission and scanning EM are routinely used to characterize the physico-chemical properties of NPs, including their shape, size and surface characteristics. The reader is referred to excellent reviews outlining the various EM techniques available and preparation protocols required 41. With regards to the study of the EPR effect, EM complements the other *in vivo* imaging technologies described here by providing direct information about NP cellular and subcellular localization (in the context of organelles and membranes), as well as the mechanisms of NP tumor extravasation (a notable example being NP extravasation as a function of tumor endothelial cell permeability 42 - which is implicated as a main driver of the EPR effect), at the nanometer resolution range. EM enables visualization of individual NP uptake events (albeit at fixed time points and *ex vivo*), provides insights to their uptake mechanism, and subsequently their intracellular fate 43. Several technical factors should be considered when EM is applied to NP visualization. For example, while inorganic NPs such as gold can be visualized using traditional sample preparation techniques, polymeric NPs, such as poly(lactic-*co*-glycolic acid) (PLGA) NPs, may not survive such preparation and offer relatively poor inherent contrast. Other preparatory approaches, such as cryo-sectioning and metallic coating can improve PLGA NP visualization 41. Given that EM samples are laborious to prepare and limited in their field of view, correlative approaches that use optical microscopy to precisely target the tissue of interest can also be extremely useful to maximize the information obtained from the EM sample 44.

#### 2.2.2. Tissue clearing for intact optical imaging

**Figure 3:**
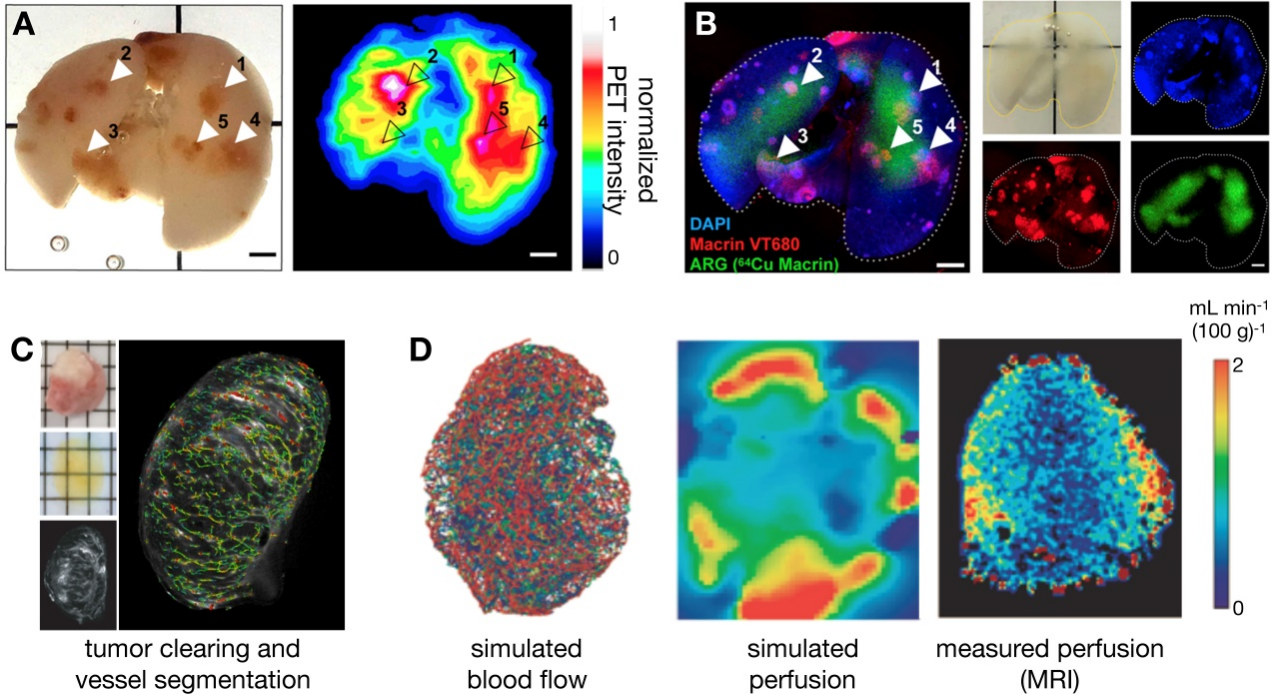
** Optical tissue clearing enables cross-modal probe validation, *in vivo*-*ex vivo* correlation and whole-tissue physiological modeling. (A-B)** ^64^Cu and near-infrared fluorophore (VT680) conjugates of Macrin, a polyglucose NP that targets tumor-associated macrophages (TAMs), were co-injected into mice bearing disseminated lung adenocarcinoma. Radioisotope distribution within the lung was detected by *ex vivo* PET (A) and autoradiography (ARG), and were correlated to the optical signal, which was obtained after tissue clearing (B). This confirmed that *in vivo* PET imaging of ^64^Cu-Macrin corresponded to selective optical-Macrin uptake within TAMs in lung tumors (example lesions highlighted with arrows, scale bar = 2 mm; Adapted with permission from 30, copyright 2018 ACS Publications). **(C-D)** d'Esposito et al. used segmented tumor vascular distributions (labeled with fluorescent lectin) from cleared tumors to simulate blood flow, interstitial fluid pressure and tumor perfusion, showing heterogeneous tumor distributions of these parameters. The modeled perfusion was compared to experimental measurements observed *in vivo* with arterial spin labeling (ASL)-MRI (Adapted with permission from 46, copyright 2018 Springer Nature).

Apart from traditional histology, EM and immunofluorescence techniques, several other *ex vivo* imaging developments show promise for the study of NP pharmacology. In particular, tissue clearing techniques render tumor-bearing organs optically transparent while retaining fluorescence signals, thus allowing complementary assessment of NP distribution at higher spatial resolutions and at greater penetration depth than possible *in vivo*. Intact tissues can be imaged in isolation, or whole animals can be optically cleared to assess systemic biodistribution without the need for embedding or sectioning, thus preserving important spatial distribution information 30, 45, 46. Moreover, the optical signal from metallic materials (such as certain gold NPs) can be amplified within optically cleared tissues, allowing the assessment of low level or small NP uptake 47. Imaging signals from cleared tissues have been successfully matched with *in vivo* imaging datasets for cellular-level interpretation of macroscopic signals. For example, Kim et al. showed good concordance between the polyglucose-NP, Macrin, in cleared tumor-bearing lungs with ^64^Cu-Macrin as imaged by PET 30 (**Figure 3A, B**). More specifically, this analysis revealed that high Macrin-PET signal correlated with the selective uptake of Macrin into tumor associated macrophages (TAMs), and that its bulk accumulation as visualized by PET was thus correlated with TAM density more than tumor size or other features 30. In other work, high resolution detail of intact tumor microvasculature from cleared tissues was used to guide models of contrast agent uptake and interstitial pressure 46 (**Figure 3C, D**). d'Esposito et al. demonstrated that distributions of model-derived tumor perfusion based on these vascular maps correlated with *in vivo* MRI measurements of perfusion (as measured with arterial spin labeling) and were able to predict uptake patterns of MRI-contrast agents. Furthermore, tumor-specific perfusion and interstitial fluid pressure patterns could be predicted after administration of the vascular disruptive agent and combretastatin A1 prodrug, OXI4503, which is relevant to understanding the EPR effect and NP delivery in tumors 46.

#### 2.2.3. Multiplexed approaches

In addition to whole-organ imaging with tissue clearing approaches, there has been accelerated improvement in multiplexed tissue imaging technologies for examining histological and/or optically cleared tissue sections. Immunofluorescence has been matched with IVM data as a way to provide immunologically- and molecularly- defined context to *in vivo* tumor imaging. Approaches to achieve this include an IVM fiducial marking strategy that allows cryosections to be correlated with IVM results 48, and the use of near infrared branding and microCT of the tumor to co-register IVM imaging with 3D electron microscopy 49. Recently, Hong et al. demonstrated the feasibility of a dual marking hydrogel system containing tantalum and india ink that could locate tissues of interest with *in vivo* CT and during endoscopy or surgery 50. These types of complementary, correlative analyses will only increase in power as new approaches such as image-cycling 51, mass spectrometry imaging, *in situ* sequencing, and spatial transcriptomics become more widely accessible 52. With respect to the EPR effect, such techniques may eventually allow relationships between NP delivery and functional EPR and TME properties to be spatially mapped to in-depth molecular characterization of neighboring tumor and stromal cells.

### 2.3. Quantitative clinical/translational imaging

#### 2.3.1. Clinical intravital microscopy

While optical microscopy is currently used predominantly in the laboratory setting, IVM setups are being developed for clinical experimental use 53, allowing possible direct clinical translation of the techniques described above. Feasibility of these setups has been demonstrated in endoscopic and intraoperative settings 54. To date, the most direct application of clinical IVM has been to identify regions of dysplasia in upper gastrointestinal and bladder malignancies, with the aid of intravenous fluorescein and fluorescently-labeled tumor targeting antibodies 55. In one example, intraoperative IVM performed on melanoma lesions found that the microvasculature in up to half the lesions imaged did not appear functionally perfused *in vivo*, as detected by fluorescein. Furthermore, *in vivo* tumor vessel diameters were on average double the size of those measured from immunohistochemistry 56. Such findings highlight that clinical IVM can provide important information with implications for NP delivery and may complement findings from pathology studies.

#### 2.3.2. Clinically relevant cross-sectional imaging

Although lower in spatial resolution, non-invasive imaging modalities such as PET and MRI complement IVM by allowing three-dimensional whole-body assessment of NP biodistribution, TME characterization and tumor treatment response. Importantly, these techniques can be directly translated for human studies. Routine clinical cancer imaging to date mainly focuses on anatomic tumor delineation (MRI or computed tomography, CT) and evaluation of tumor metabolism (^18^F-fluorodeoxyglucose PET, FDG-PET). New molecular imaging agents and technical developments aim to provide additional insights in systemic NP kinetics as well as translating microscopy findings into clinically relevant assays.

#### 2.3.3. Positron emission tomography

PET is a highly sensitive imaging modality that, when combined with attenuation correction information provided by CT or MRI in combined PET/CT and PET/MRI systems, enables quantitative analysis of systemic biodistribution. Studies have demonstrated the utility of PET for assessing NP kinetics and dynamics 30, 57, 58. Radioisotopes with half-lives commensurate with the extended kinetics of NPs exist 30, 58 (e.g. ^64^Cu t1/2 = 12.7 hours and ^89^Zr t_1/2_ = 78.4 hours) and have been used to directly label NPs, including inorganic nanorods 59, liposomes 60, dextran-based polyglucose particles 30, along with cyclodextrin-based NPs 61. Many small molecule drugs can be radiolabeled and coupled with alternatively radiolabeled nanocarriers (for example with ^111^In labeled liposomes for single photon emission computed tomography, SPECT, imaging). This strategy enables simultaneous multimodal imaging of nanocarriers and their drug payloads 62, analogous to what has been done in IVM studies 25, 31, 32, 36. Furthermore, new PET imaging agents are being developed to interrogate TME features including vasculature 63, tumor hypoxia 64, extracellular matrix (ECM) 65 and immune cell-subsets 66. New conjugation techniques now exist that allow antibodies and NPs to be dually labeled with optical and radioisotopes, enabling correlative multimodal assessments of NP uptake 30, 58, 67. The availability of small animal PET/CT and PET/MRI systems can also allow cross-species comparison of the same agent. For example, Lee et al. recently assessed the biodistribution of a ^64^Cu-labelled liposomal doxorubicin (MM-302) in murine preclinical studies 60, 68, guiding the dosage and kinetic assessment in subsequent clinical studies 57, 69. Most recently, the development of total body PET/CT systems with sensitivities up to 40 times that of standard clinical PET/CT scanners will allow detection of radio-labeled-NPs for longer time points and at lower concentrations, further providing systemic kinetic information not previously accessible by current clinical scale modalities 70.

#### 2.3.4. Single photon emission computed tomography

SPECT is a scintigraphic technique that makes extensive use of NP in routine clinical practice, including ^99m^Tc-sulfur colloid (particle size of 253 ± 192 nm when unfiltered 71) and ^99m^Tc-macroaggregated albumin (MAA; 10-90 μm in size). ^99m^Tc-MAA is routinely used clinically to predict the tumoral distribution of radioembolization beads. While several past studies have used SPECT to examine the EPR effect 71, and the technique can be relevant for preclinical studies 72, we anticipate that the increased sensitivity and spatial resolution offered by PET will make it a more promising modality for clinical NP assessment in the future.

#### 2.3.5. Magnetic resonance imaging

MRI provides high spatial and soft tissue contrast information compared to PET, enabling the assessment of the TME in conjunction with NP delivery. A number of MRI-visible nanoprobes have been developed; most of these rely on modulation of proton T_1_ and T_2_ relaxation times using iron oxides 73-78, gadolinium 79 and manganese 80, or other nuclei such as ^19^F 81, and have been incorporated into nanomaterials to achieve MRI-visible contrast. Conventional MRI has relatively low sensitivity; hyperpolarized MRI can conceivably boost the MR signal 10,000-fold, improving NP sensitivity. Several hyperpolarized nanomaterials have been developed using silicon NPs and nanodiamonds 82, 83, with *in vivo* imaging demonstrated to be feasible in murine tumor models.

To date, ferumoxytol (FMX) remains among the most extensively studied MRI-visible NP that has been translated for clinical studies. FMX is a carboxymethyl dextran coated iron-oxide particle that is FDA-approved for the treatment of iron deficiency, and has been examined for its ability to efficiently accumulate in myeloid phagocytes including TAMs 25, 78. Several preclinical and clinical studies have demonstrated the utility of FMX (diameter ~20nm) to predict uptake of therapeutic NPs. FMX holds promise as a viable MRI-based NP companion diagnostic, and has been shown to correlate to TAM-levels as assessed by pathology 84. One potential issue of current FMX imaging is that it is based on negative susceptibility (T_2_) contrast, which can be challenging to interpret in the context of tumor necrosis or other areas where susceptibility artifacts are of concern, such as in the lung or pelvis. New technical developments enabling positive T_1_-based contrast of iron oxide with ultrashort TE or other sequences 85 will facilitate visualization of FMX and likely increase its adoption in clinical imaging studies.

As with PET and IVM, robust quantitative assessment of the MRI signal (i.e. T_1_ and T_2_ mapping) would allow comparison between individual studies and also with IVM results. This has been challenging to implement with *in vivo* studies to date, given the long duration required and technical challenges (e.g. B1 field inhomogeneity) involved for robust T_1_ and T_2_ relaxation time imaging assessment. Approaches based on compressed sensing 86 and MRI fingerprinting 87 promise to allow quantitative T_1_, T_2_ and proton density mapping to be performed within clinically viable imaging timeframes. These parametric maps can be applied for quantitative assessment of localized NP uptake and pharmacokinetic assessment of TME parameters. For instance, vascular permeability using dynamic contrast-enhanced MRI (DCE-MRI) relies on robust concentration vs. time curve assessments at high time resolutions 88, and accurate assessment of local FMX tumor concentrations using T_2_ -weighted sequences require robust T_2_ relaxation time measurements.

#### 2.3.6. Combined modalities and future developments

Multimodal clinical imaging can provide a correlative assessment of NP uptake with other aspects of the TME. Ultrasound/MRI, SPECT/CT, PET/MRI and PET/CT systems exist and are being increasingly adopted for NP distribution studies. Apart from providing anatomical information, CT angiograms have been used to create vascular masks from which realistic tumor vasculature maps can be used to model NP delivery 14. CT-visible NPs with extended kinetics have also been developed to assess both the vascular and lymphatic systems 89. DCE-MRI can also provide similar information, but care needs to be taken to ensure that the contrast agent used for this purpose has pharmacokinetic properties commensurate to the NP of interest 90.

Other promising and emerging translational imaging modalities are also being used to understand NP uptake. Ultrasound can be used to visualize sonoluminescent NPs; additionally, focused-ultrasound techniques can modulate the EPR and effect localized drug release 91. Magnetic particle imaging (MPI) in particular is showing great potential in preclinical studies as a highly sensitive and quantitative modality to examine the distribution and uptake of super paramagnetic NPs, and other recent reviews have described such methods in detail 92.

## 3. Using image-guided systems pharmacology to elucidate and manipulate the TME and EPR effect

**Figure 4:**
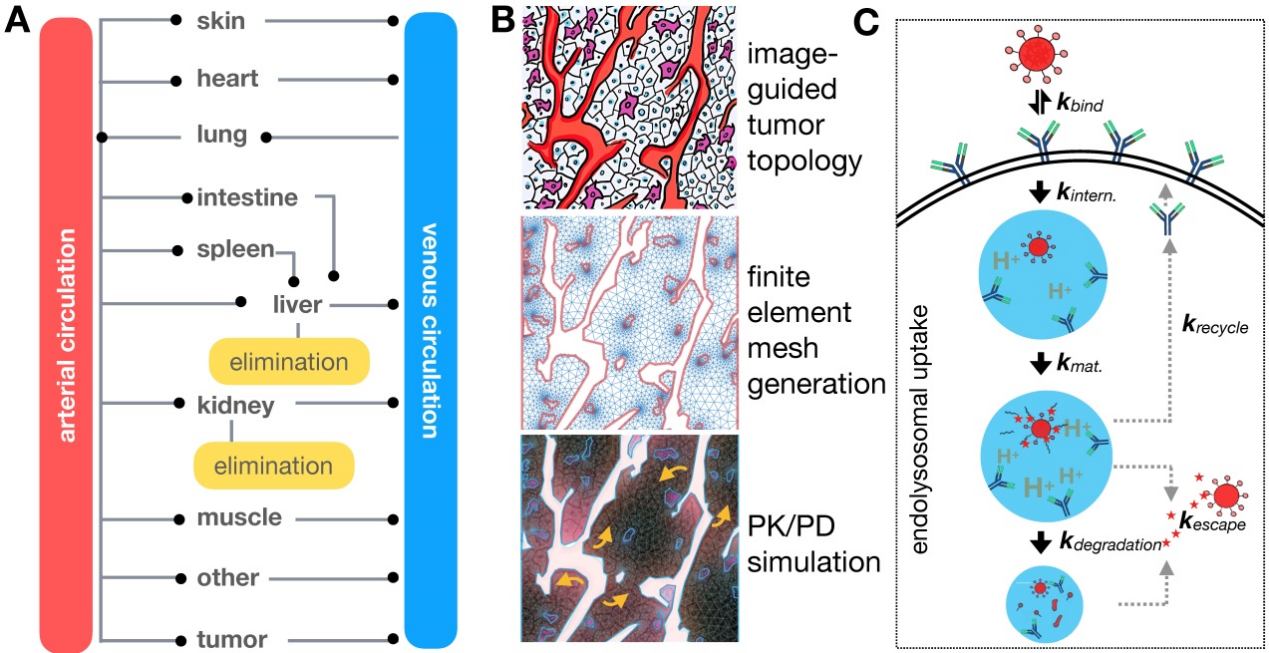
** Computational modeling captures EPR determinants at the organismal, tissue, and single-cell level.** (**A**) Physiology-based pharmacokinetic (PBPK) models with multiple tissue/organ compartments aid in understanding systemic NP biodistribution and clearance kinetics. (**B**) Systemic pharmacokinetics are coupled with localized tumor tissue models of NP-TME interactions, here represented as a finite element reaction/diffusion model derived from anatomical and functional IVM data (Adapted with permission from 25, 32, copyright 2015, 2017 AAAS). (**C**) Interactions of individual NPs with cellular surface and subcellular structures can also be examined, here showing NP uptake by cells via receptor-mediated endocytosis (Adapted with permission from 117, copyright 2014 Elsevier).

TNP delivery and action is governed by a balance of systemic biodistribution and clearance (**Figure 4A**), extravasation and elimination locally at the tumor site, interaction with tumor cells and the TME (**Figure 4B, C**), and ultimately their impact on cancer cell killing. These processes span over six orders of magnitude spatially 11, thus making it a challenge to implement a single pharmacological model that is computationally practical and biologically relevant across the entire spatiotemporal spectrum. Consequently, multiple models spanning different temporal and spatial scales are typically used to understand particular aspects of NP behavior (**Table 1**), although multi-scale frameworks have successfully bridged length-scales in some applications 13. TNP kinetics and dynamics are typically considered more complicated than their small-molecule therapeutic counterparts. For instance, compartmental modeling is suited for understanding NP systemic pharmacokinetics (**Figure 4A**), whereas a finite element reaction/diffusion model derived from anatomical and functional IVM data is more suited for understanding localized NP extravasation from tumor vasculature (**Figure 4B**). By design, NP kinetics comprise a multicomponent process defined by kinetics of the NP vehicle, payload encapsulation, drug release and carrier breakdown. Thus, many assumptions made by pharmacokinetic models of small molecule drugs do not capture the spatial and temporal heterogeneity that drive NP behavior. Accordingly, models developed for NP kinetic analysis need to account for these differences, and imaging data is vital to ensure the fidelity of the models developed for this purpose. Several excellent reviews have examined the role of mathematical modeling in cancer nanomedicines 10-12. Building on this literature, we focus in particular on how imaging has been combined with computational modeling at multiple scales for the study of TNP pharmacology.

### 3.1. Compartmental modeling of systemic TNP pharmacokinetics

Once NPs are injected into the bloodstream, they are transported via blood to the tumor and organs. How this systemic biodistribution manifests is most commonly modeled as a set of interconnected compartments, with simple models describing the system by two compartments: a central compartment incorporating blood pool and other highly perfused organs, and a peripheral compartment depicting less perfused or slowly equilibrating tissues such as tumor, fat and muscle 11. First order kinetics are often assumed for transfer between the compartments and for elimination. Physiological-based pharmacokinetic models (PBPK) build upon this by adding anatomically based compartments connected with individual blood flow rates (**Figure 4A**). Each compartment's parameters can be populated individually, for example, through imaging, and compartments can be further sub-divided into vascular, interstitial and cellular components. First order kinetics are again often assumed, resulting in a system of first order differential equations (ODEs) to model the concentration time course of NPs in tissues and plasma. Translational imaging modalities (e.g. PET, SPECT) are ideal for providing information to fit such models, given that their whole-body field-of-view can provide concentration vs. time information for multiple tissues and organs at once 72. However, factors such as time resolution and partial volume effects can profoundly affect accurate assessment of the concentration time curve. Overall, these models have been especially useful in i) understanding systemic TNP clearance, primarily by the mononuclear phagocyte system (MPS) of the liver and spleen 93; ii) interpreting and predicting differences in systemic pharmacokinetics across preclinical animal models and in patients 94; and iii) in analyzing and predicting variable systemic pharmacokinetics across patients receiving TNP treatment, for instance as may be impacted by body mass index 95, systemic MPS activity 96, and the presence of anti-PEG antibodies 97. It has been noted that differences in MPS activity across animal species has complicated PBPK scaling across animal models compared to small-molecule drugs, which further highlights the need for systems-level perspective when translating TNP pharmacology from preclinical models to patients 96.

### 3.2. How does the TNP administration route impact its systemic kinetics?

In addition to the above examples, image-guided pharmacokinetic models have been useful for interpreting and predicting how the behavior of NPs can depend on their route of administration. While the majority of TNP studies have focused on intravenous NP delivery, multiple studies and clinical applications have explored other routes of administration, including injection into the intraperitoneal cavity 72, arteries (such as used for hepatic chemo- or radio-embolization procedures) 98, subcutaneous tissue 99, and the bulk tumor mass directly 100. Inhaled 101 and topically applied formulations are also relevant. For example, when two additional compartments simulating the peritoneal cavity and lymphatic drainage for intraperitoneal injected NPs were added to a PK model of mesoporous silica NPs 72, it was found that the chief impact of intraperitoneal compared to intravenous injection was only a slight delay in systemic PK as the NPs traversed through the lymphatic system 72. These results contrast with clinical observations that solvent-based intraperitoneal chemotherapy can exhibit superior efficacy in the treatment of disseminated ovarian cancer 102, suggesting context-dependent distinctions between intraperitoneal and intravenous administration. As a less nuanced comparison, intratumoral injection of NPs can have a more substantial impact on systemic NP biodistribution. Using transmission electron microscopy, Giustini et al. mapped the kinetics of intratumoral-injected iron oxide NPs (100-130 nm diameter) in murine breast cancer xenografts 103, showing that almost all NPs were taken up by tumor cells by 4 h post injection and cleared from the extracellular space, aggregating within cytoplasmic vesicles. Similarly, intratumorally injected radioactive ^103^Pd:Pd@Au-PEG NPs have been found to aggregate in cytoplasmic vesicles by 24 h post intratumoral injection in a prostate cancer model 104. Scintillation counting showed that 92% of the injected dose remained within the tumor 8 days post-injection (compared to <15% in the liver). MicroCT, optical and electron microscopy imaging obtained in this study were applied to macro- and microdosimetry mapping within the tumor using Monte Carlo simulations, finding that Au-PEG NPs were able to deliver a higher localized dose compared to standard brachytherapy seeds. Imaging and corresponding modeling suggests that generation of reactive oxygen species by radiation is more dominant than direct DNA strand breaks with this mode of NP delivery, given the relative lack of uptake in the nucleus. Ongoing studies continue to examine how material designs influence retention of intratumorally-injected nanomaterials (including viruses) and their trafficking to draining lymph nodes 105. Taken together, these studies highlight the potential of alternative injection routes to affect NP and payload drug/radiation distribution within the tumor.

### 3.3. How do intrinsic physicochemical properties of TNPs impact their delivery and action?

#### 3.3.1. Size, shape and surface properties impact margination, transport, and cellular uptake

**Figure 5:**
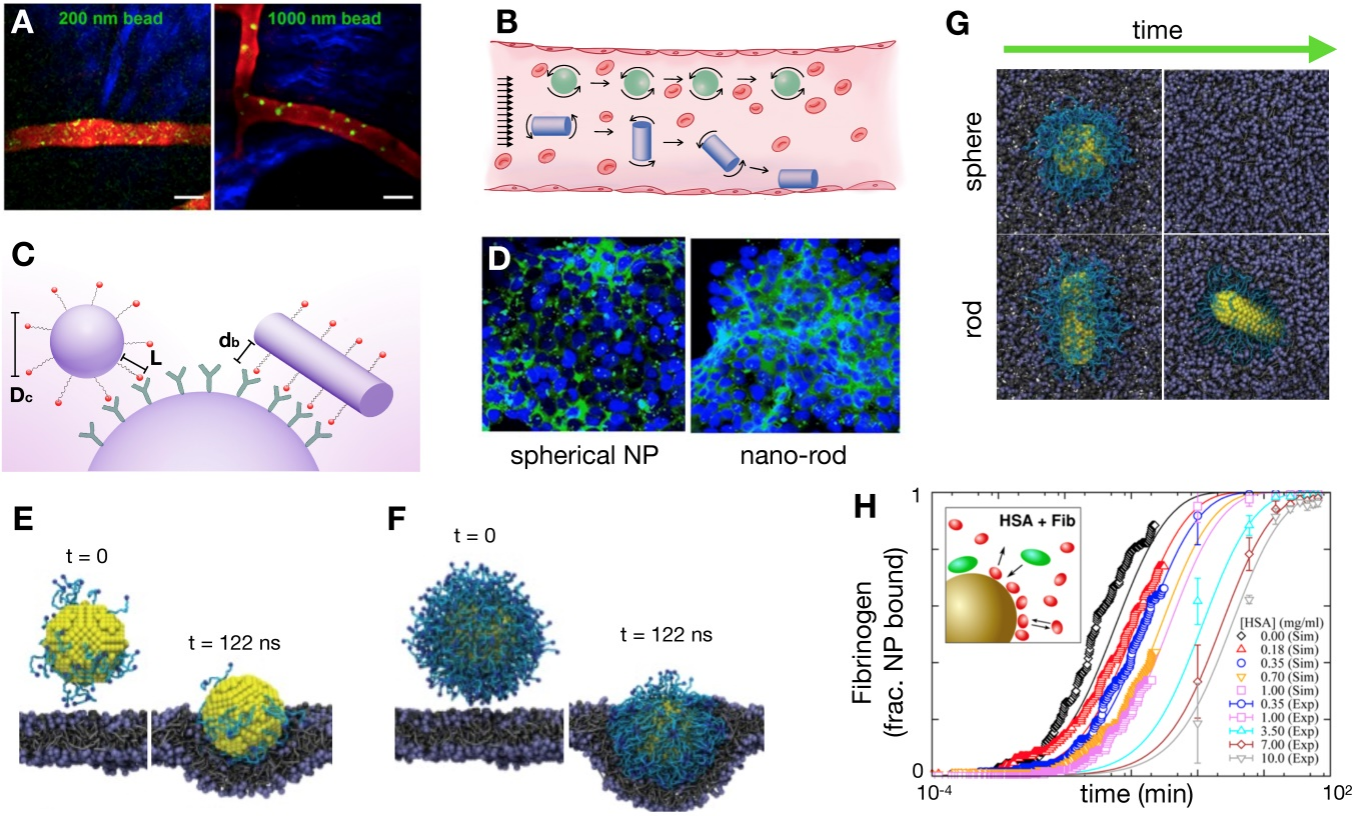
** NP size, shape and coating impact kinetics at different spatial scales.** (**A**) IVM quantifies spherical polystyrene beads in the postcapillary venule of mouse ears, showing that larger NPs marginate in the blood vessels more than smaller NPs (Adapted with permission from 107 copyright 2013 Springer Nature). (**B**) NP shape impacts vessel margination, as variable forces and torques exerted on rods under flow promote drift towards the vessel wall, where they may bind to receptors or extravasate through endothelial gaps (Modified from 106). (**C**) Shape, ligand length, and polymer flexibility all contribute to the active fractional area of a nano-carrier (AFAC). For a sphere, the AFAC is defined as (L-d_b_)/D_c_, where L is the length of the ligand, d_b_ is the binding distance between the nanoparticle and the receptor, and D_c_ is the diameter of the nano-carrier (Modified from 106). (**D**) Shape dependent NP uptake is exemplified here with increased anti-HER2 trastuzumab-coated nano-rod accumulation in HER2+ breast cancer cells compared to spherical NPs (Adapted with permission from 120, copyright 2013 National Academy of Sciences, U.S.A.). (**E-F**) Dissipative particle dynamics modeling simulates the effect of PEGylation density (blue-green) on the NP surface and the effect of shape (**G**) on internalization dynamics. Increased PEGylation and spherical shape promote internalization (Adapted with permission from 121, 123, copyright 2014, 2015 Elsevier, Royal Society of Chemistry). (**H**) Coarse-grained modeling of competitive protein adsorption onto silica NPs was used to simulate the competitive adsorption between human serum albumin and fibrinogen at differing solution concentrations. Simulations based on the non-Langmuir differential rate equation (which enabled extrapolations of findings over long time scales, > 1 h, open symbols) showed that protein adsorption is a competitive process. These findings correlated well with experimental data (symbols with error bars; Adapted with permission from 125, copyright 2016 ACS Publications).

Imaging, particularly at microscopic scale, has clarified our understanding of how factors intrinsic to NPs, including NP size, shape, molecular coating, and surface charge can affect their delivery 106. For instance, particle size can impact margination of NPs in tumor vasculature. As observed using an IVM-guided finite element model, smaller particles tend to be evenly distributed throughout the vessel diameter and remain in circulation, whereas larger particles are more likely to marginate to the vascular walls, increasing their likelihood of extravasation 107 (**Figure 5A**). Shape can also impact NP margination, with non-spherical shapes partitioning toward the vessel wall at a higher degree compared to spherical particles 108 (**Figure 5B**), potentially resulting in increased extravasation in vessels with relatively smaller (100 nm) pore sizes 109.

The surface charge of NPs can profoundly impact NP delivery, cellular uptake and cytotoxicity 110. For instance, phagocytic cells have a predilection for charged NPs, which can limit the NP bioavailability at the tumor site due to efficient MPS clearance. Extensive work has been done to optimize NP surface charge properties to minimize this effect 111. Surface charge can also impact the degree of tumor penetration, with both cationic and anionically charged NPs showing improved extravasation and tumor penetration depending on the TME content and tumor stage in some cases 112, 113. Positively charged cells show generally better uptake in non-phagocytes, and also can exhibit increased cytotoxicity due to membrane damage. However, such positive surface charge does not always confer cytotoxicity, for instance as seen with chitosan polysaccharide-coated PLGA NPs 114. Molecular dynamics (MD) modeling combined with EM can guide rational design of NPs. For example, Mendes et al studied the effect of cationic surfactants upon uptake of ultra-small nanostructure lipid carriers in glioblastoma multiforme. MD simulations found that monomeric serine-derived surfactants resulted in the improved interaction with lipid bilayer structures, suggesting improved membrane permeation and hence cellular uptake; this was corroborated with NP uptake assessment using EM and optical imaging performed in the same study 115.

Internalization of NPs into tumor and phagocytic cells can occur via multiple and non-mutually exclusive pathways including receptor-mediated caveolin and clathrin dependent endocytosis, macropinocytosis, and phagocytosis 116. With respect to receptor-mediated uptake, Sorrell et al. developed a kinetic model accounting for the rate of change of unbound, bound and internalized NPs, and concluded that NP uptake depended on the number of receptors concomitantly engaged by the particle 117. The impacts of NP shape, stiffness and surface coating upon cell internalization have also been studied and computationally modeled 116. For instance, Gao et al. and Decuzzi et al. examined the shape dependence of NPs to affect their endocytosis 118, 119. These studies found an optimal range of aspect ratio values for ellipsoid particles to maximize internalization, with rod-shaped NPs demonstrating a more favorable geometry for ligand binding (**Figure 5C**). Along similar lines, nano-rods coated in the anti-HER2 antibody trastuzumab were observed to internalize into cells more than spherical NPs in a BT-474 murine breast cancer model, supporting computational findings (**Figure 5D**) 120. The amount of PEGylation can impact NP internalization. Dissipative particle dynamics methodology can be used to model the hydrodynamic interactions of NP (**Figure 5E, F**), with results showing that increased PEGylation density surrounding gold NPs lead to more efficient endocytosis 121; similar results have been observed experimentally 122. Through computational simulation, PEGylated spherical NPs have also been found to endocytose most efficiently compared to rod, cube and disc shapes 123 (**Figure 5G**). Taken together, the above findings collectively suggest that non-spherical geometries facilitate NP extravasation and cellular binding, especially when functionalized with receptor binding ligands, while sphericity and PEGylation of NPs can promote their cellular internalization. In the future, these approaches are aptly suited to examine impacts of the protein corona — that is, the adsorbed and associated proteins surrounding a NP — on NP binding and uptake.

#### 3.3.2. How does the protein corona alter TNP properties?

Several models have been developed to examine the process of protein corona formation, including those that describe the effect of NP surface shape, size and charge 124, the time evolution of the protein corona in the presence of different types of protein 125, and how the protein corona changes as the NP moves between biological compartments 126, including as NP crosses the blood brain barrier 127. For instance, molecular simulations based on the non-Langmuir differential rate equation showed that adsorption of fibrinogen and human serum albumin on the surface of silica-NPs are competitive and is concentration dependent (**Figure 5H**) 125. The additive effect from these processes can potentially impact overall NP biodistribution. The protein corona can alter the apparent NP size and charge, which can affect extravasation and interactions with the MPS 128. Certain proteins that are adsorbed onto the NP surface may be ligands for membrane receptors, which can impact NP cellular internalization 129 and immune recognition 130. Importantly, the protein corona may interact with the drug payload, affecting its release. For example, protein interactions with nucleic acids such as siRNA may result in unintended release of the nucleic acid 131. Presence of the proteins around the NP can alternatively reduce drug release, such as seen with albumin-bound paclitaxel 132. In future studies, image-guided QSP promises to be useful for quantifying the functional impacts of NP protein coronas, and greater comprehension of protein corona behavior can in turn guide the refinement of QSP models more generally.

### 3.4. Molecular targeting improves TNP delivery in some but not all contexts

**Figure 6:**
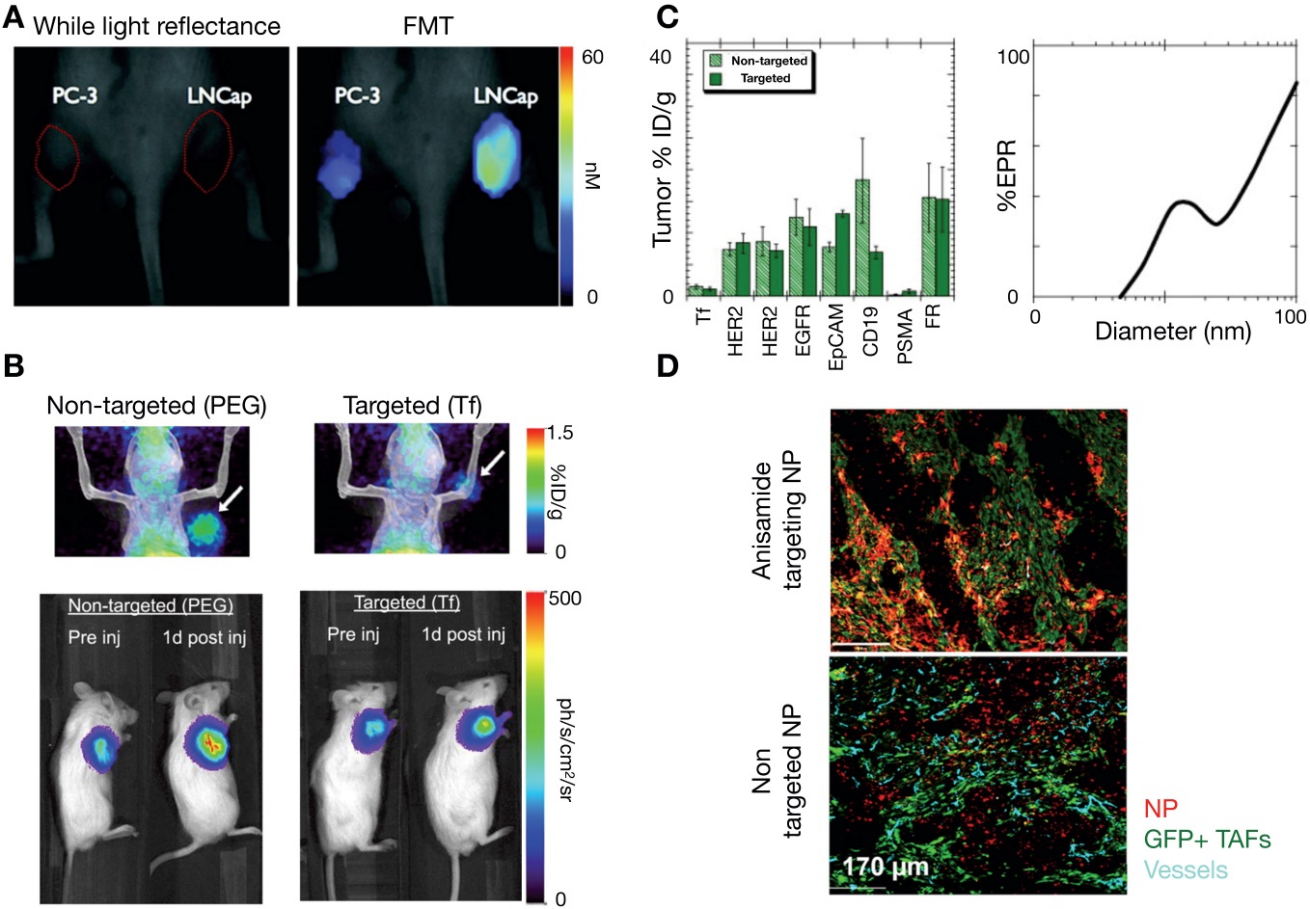
** Effect of active targeting on NP delivery.** (**A**) Active molecular targeting of NPs has been demonstrated as a viable strategy to increase tumor uptake. Here, hepsin-targeted NPs (~40 nm) preferentially accumulate in hepsin-expressing LNCap prostate xenografts compared to hepsin-negative PC3 tumors (Adapted with permission from 135, copyright 2008 AACR). (**B**) Using compartmental modeling, Bartlett et al. showed that the benefit of active targeting lies in increasing cellular uptake. Transferrin-targeted NPs (~100 nm) showed similar overall tumor uptake to non-targeted particles in a Neuro-2A tumor model, as seen by PET (top), but the effect of the siRNA payload was more pronounced with targeting, as demonstrated by siRNA silencing of luciferase expression (bottom; Adapted with permission from 136, copyright 2007 National Academy of Sciences, U.S.A.). (**C**) Computational modeling by Wittrup et al. support this finding from *B*, showing bulk tumor accumulation of larger particles (~100 nm) depends mostly on passive EPR effects (Adapted with permission from 137, copyright 2012 Elsevier). (**D**) Miao et al. experimentally demonstrate the complex trade-offs inherent with NP targeting: anisamide-targeted and non-targeted small (18 nm) lipid-coated NPs (red) were injected into mice with tumors containing anisamide-expressing tumor associated fibroblasts (TAFs). At 16 h post-injection, non-targeted NPs penetrated further into the GFP-negative tumor region than targeted-NPs, which was attributed to the 'binding site barrier' as targeted-NPs become retained by targeted GFP+ fibroblasts near vessels (Adapted with permission from 139, copyright 2016 ACS Publications).

In addition to molecular targeting of tumor vasculature 133, active molecular targeting of tumor cells themselves by NP surface functionalization with specific ligands has been explored as a means to improve NP delivery 134. Early studies showed the promise of this approach, highlighting increased uptake of tumor cell-targeting NPs compared to passive targeting NPs. For example, Kelly et al showed that hepsin-targeting peptides conjugated to cross-linked iron oxides NPs improved NP tumor accumulation in hepsin expressing prostate tumor xenografts compared to non-targeted NPs 135 (**Figure 6A**). Furthermore, active targeting has been demonstrated to enhance therapeutic efficacy by improving NP cellular uptake. For example, dynamic PET imaging data from a ^64^Cu-labelled and transferrin-targeted cyclodextrin NP, applied to a compartmental model of tumor uptake, showed that bulk tumor targeting is not affected by ligand functionalization 136. Yet in the same study, bioluminescence imaging demonstrated that transferrin-targeted NP containing siRNA attenuated tumor cell luciferase expression better than non-targeted NP (**Figure 6B**). Taken together, these findings suggest that active targeting impacted tumor cell uptake, but not bulk tumor accumulation. Further modeling studies have highlighted this complexity 137. Using compartmental modeling simulations, Wittrup et al. confirmed that passive NP targeting via the EPR effect is the predominant process affecting NP tumor uptake, especially for larger NPs (> 50 nm, **Figure 6C**). However, similar to antibodies, tumor penetration of targeted NPs can be tempered by the 'binding site barrier', whereby NPs bind to targeted cells near vessels, limiting further tumor penetration. A reaction-diffusion model to explore binding site barriers to NP tumor penetration found that the majority of NPs do not penetrate far from the vascular bed 138, and that delaying the binding of NPs to target cells can improve tumor penetration. This has been observed in recent imaging studies, for instance in the molecular targeting of anisamide-expressing fibroblasts: anisamide-targeted NPs were observed to bind to anisamide-expressing fibroblasts near vessels, whereas non-target NPs were able to penetrate further into the tumor (**Figure 6D**) 139. For future studies, understanding the interplay of the NP binding site barrier effect with strategies that alter tumor perfusion and IFP 68 will be especially useful.

### 3.5. Intracellular processing and drug pharmacodynamics

Once internalized, NPs often traffic through early and late endosomes, and subsequently are degraded in lysosomes, with their contents released to cells or exported (**Figure 4C**). The fate of NPs within the endo-lysosomal pathway can impact their efficacy. Using optical imaging, Seynhaeve et al. and others noted that doxorubicin associated with liposomal formulations can be substantially sequestered in the lysosomal compartment, as opposed to its intended target of DNA in the nucleus 140, 141. Similarly, polymeric micellar TNP have been observed to accumulate within the endo-lysosomal pathway 31, 36, 94. Although a fraction of co-encapsulated therapeutics are found in endo-lysosomal compartments after NP uptake, both *in vitro* and IVM experiments have demonstrated how small-molecule drug payloads can gradually release from this sequestration to act on cells that have accumulated TNP, and cells in proximity as well 4, 31, 94. The problem of endosomal escape is especially acute for nucleic acid delivery, and imaging has been key in visualizing subcellular distributions of NPs and their nucleic acid payload, for instance showing delivery of DNA and RNA to the nucleus and cytoplasm, respectively, in a gold-NP CRISPR formulation 142. Computational models can be useful in understanding these processes 143. For example, Mihaila et al. developed a system of ODEs to describe the delivery processes of different lipid NPs containing siRNA, from particle endocytosis to the association of siRNA with the RNA induced silencing complex 144. This modeling found that rates of endosomal escape were among the major differentiators between NP efficacies compared to other processes such as cellular NP uptake.

Success of NP-drug design is measured ultimately by its therapeutic efficacy, and several models have incorporated cell kill into their analysis. Pascal et al. developed a mathematical model that evaluated the delivery of nano-formulated doxorubicin to hepatocellular carcinoma tumor cells, and concluded that improved efficacy achieved by TNP could largely be attributed to enhanced cellular uptake rates of TNP compared to solvent-based formulations, particularly in cancer cells overexpressing the drug efflux pump P-glycoprotein (MDR1) 145. In another study, van de Ven et al. developed a model of NP delivery to the tumor vasculature, with subsequent drug release to the surrounding tissue over 4 days. They found a non-linear relationship between drug delivery and cancer cell killing, in part attributable to the time scale discordance between drug diffusion and uptake compared to the process of cell death. Consequently, the study emphasized the need to design TNP strategies that sustain drug delivery to the tumor over time 146. Linking such pharmacodynamic models to models of delivery and to traditional and image-based methods of efficacy evaluation (i.e. tumor shrinkage) should continue to be advanced in the future. There has been an expansion in the ability to monitor downstream drug impacts at the single-cell level optically and by IVM. Fluorescent protein and luciferase expression have been longstanding tools to monitor nucleic acid therapeutics. Relevant to cytotoxic chemotherapies, fluorescent imaging reagents have been used to examine cell-cycle 147, nuclear fragmentation and apoptosis 147, binding of microtubule-targeted drugs 148, and DNA damage response 149: all of this information being obtainable using IVM and at the single-cell level. For immune-targeted therapies, IVM readouts have included quantification of immune infiltration and migration 150, along with pro-inflammatory cytokine secretion 151 and single-cell level expression of macrophage-polarization markers 152. Such quantitative, dynamic, and high-resolution imaging has the potential to guide modeling efforts that capture not just heterogeneous drug delivery, but also variable and often stochastic drug responses in order to better understand PK/PD relationships at a single-cell level.

### 3.6. How does the balance of vascular perfusion and permeability impact TNP penetration?

Given that the chief basis of the EPR effect relies on the unique properties of tumor vasculature, a large number of studies have focused on understanding the interactions of NPs with vasculature 153. IVM is especially useful at this scale, since it enables visualization of intratumoral vascular perfusion at a high spatial and temporal resolution. Dynamic imaging data obtained from these studies permit the assessment of localized flow kinetics, tissue permeability and blood volume fraction, and has demonstrated variability of these parameters within individual tumors 20. Differences in perfusion within individual tumors have been classified and correlated with differential NP extravasation 154. Perfusion variability and its impact on NP delivery has been assessed within 154 and across tumor types, for instance highlighting differences between highly vascularized renal cell carcinomas and poorly perfused pancreatic lesions 109, 155. From these studies, NPs with diameters greater than 100 nm were consistently found to penetrate more poorly in hypovascular tumors compared to smaller NPs, emphasizing the need to modulate the EPR especially in these tumor subsets.

**Figure 7:**
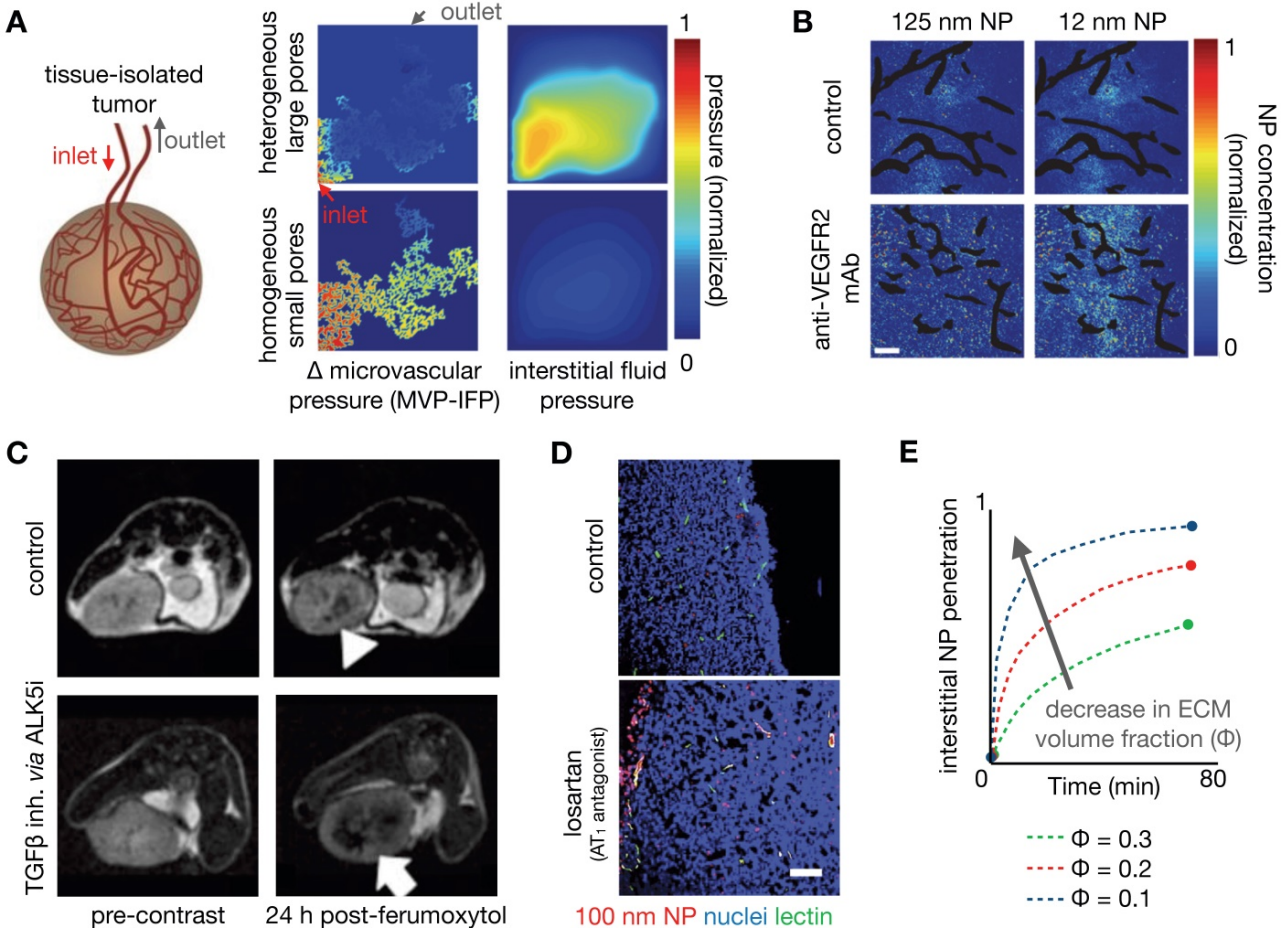
** Improving NP penetration by manipulating vascular permeability and mechanical stress. (A)** Tumor vasculature can be modeled as a percolation network defined by vessels with pores of various sizes, used in this example to understand how heterogeneous pore sizes can impact tumor perfusion and NP delivery. Large heterogeneous pores produced elevated interstitial pressures (IFP) approaching the mean vascular pressure (MVP), resulting in a diminished transvascular pressure gradient and poor drug extravasation. Small homogeneous pores, thought to mimic vasculature “normalized” by treatments such as anti-VEGF mAb, result in high transvascular pressure gradients that drive convective drug delivery (Adapted with permission from 157, 170, copyright 2012, 2017 Springer Nature). (**B**) Normalization of vasculature with therapeutic anti-VEGFR2 mAb (DC101) in mammary tumors demonstrates increased penetration of 12 nm NPs, but not of 125 nm NPs (Adapted with permission from 157, copyright 2012 Springer Nature). (**C**) Ferumoxytol-MRI quantifies improved NP penetration in a mouse mammary tumor model after treatment with an ALK5 inhibitor, which blocks TGFβ signaling (Adapted with permission from 162, copyright 2016 American Society for Clinical Investigation). (**D**) The angiotensin II type 1 receptor (AT_1_) antagonist losartan reduces intratumoral collagen, mechanical stress, and IFP, which improved penetration of model polystyrene NPs in a pancreatic cancer model. NPs are localized around perfused vessels (green) (Adapted with permission from 167, copyright 2011 National Academy of Sciences, U.S.A.). (**E**) Penetration of NPs is modeled as a function of time from entering the tumor vasculature and for varying ECM fractions (Φ), such that decreased ECM and mechanical stress resulted in improved NP penetration (Adapted with permission from 167, copyright 2011 National Academy of Sciences, U.S.A.).

As one strategy to understand the role of perfusion and vascular permeability on NP delivery, Chauhan et al. developed a model of tumor vasculature based on percolation theory 156, 157, with the vessel network comprising a series of interconnected nodes representing blood vessel segments, and with each segment containing a range of vessel wall pores of varying sizes (**Figure 7A**). In this work, blood vessel flow, transvascular fluid exchange, and interstitial transport followed Poiseuille's law, Starling's approximation, and Darcy's law respectively. Pore theory was used to calculate hindrances to diffusion and convection. Using this model, the authors demonstrated the significance of vascular normalization using anti-angiogenic therapy to improve NP extravasation, especially for small (~12 nm diameter) NPs. Frieboes et al. integrated a multidimensional tumor growth model that accounted for the time dependent development of tumor vasculature with modeling of vascular adhesion of NPs 133. Using this model, the authors assessed the relationships between tumor growth stage and NP properties in determining the spatial distribution of NPs, identifying that a balance between NP vascular affinity and flow rates within neovasculature is especially important in governing tumor NP distribution.

#### 3.6.1. Therapeutic vascular modulation improves TNP delivery

Manipulation of the tumor vasculature has been extensively studied, especially through the VEGF signaling axis. Jain et al. have highlighted the concept of vascular normalization, whereby low to intermediate dosing of anti-angiogenic therapies render abnormal tumor blood vessels less leaky. This strategy is thought to improve functional tumor perfusion and corresponding drug delivery 156, 157. As noted above, Chauhan et al. examined the effects of an anti-VEGFR2 antibody on nanoparticle penetration using computational modeling. Low dose angiogenic treatment resulted in transient decreases in vessel diameter in orthotopic mammary tumors, enabling a 3-fold increased penetration of small (12 nm) diameter NPs, but not larger particles (>60 nm, **Figure 7B**) 157. The effects of vascular pore size changes were modeled within the percolating vascular network model, suggesting that the improvement is manifested by decreased vessel pore sizes with resulting decreased IFP and thus better NP penetration (**Figure 7A**). Cabral et al. also found similar effects upon treatment with a TGF-β inhibitor 155. Building upon these findings, Jiang et al. found that extravasation of intermediate-sized (20-40nm) NPs also benefited from vascular normalization, but larger particles (>70 nm) experienced increased diffusional hindrance within the interstitium 158. Collectively, these studies have revealed that enhancement of NP uptake due to vascular normalization is both dose and time dependent, and thus relies on a "normalization window" that facilitates drug delivery 157. Normalized vasculature shows improved pericyte coverage, pruning of aberrant vessels and reduction of tumor hypoxia, acidity and fluid pressure, which results in the improved extravasation of drugs, including small NPs. At high anti-angiogenic doses, this normalization window narrows, and decreased tumor vascularization from anti-vascular effects can result in decreased perfusion and hence compromised drug delivery. Vascular normalization has been observed in patients in multiple types of cancers 159. For example, Willett et al. showed decreased vascular flow, permeability, and IFP in rectal tumors after a single dose of bevacizumab, as guided by dynamic CT and endoscopy 160. FDG uptake measurements within 2 weeks of treatment in these patients did not reveal a significant decrease, suggesting the improved efficiency of the remaining vasculature for drug/probe delivery.

Other pharmacological methods to enhance vascular permeability of NPs have also been explored. For example, treatment with tumor necrosis factor alpha (TNFα) has resulted in a 10-fold increase in radiolabeled liposome uptake, as seen by SPECT/CT 161. FMX was also shown to be sensitive to the downstream effects of inhibiting the type I TGF-β receptor, activin-like kinase 5 (Alk5), which can enhance vascular permeability, decrease IFP, and improve tumoral NP accumulation (**Figure 7C**) 162. Overall, the described studies highlight the potential of modulating vascular permeability to improve TNP delivery, with NP size and anti-angiogenic therapy dose being important factors impacting its efficacy. However, given the transient nature of the 'normalization window', these strategies also present a translational challenge: how do we identify the optimal window for individual patients? DCE-MRI and FMX imaging provide possible companion diagnostic options to identify normalized vasculature 163, and the availability of reliable imaging quantitation of vascular permeability will be key to the success of these imaging tests. Alternatively, strategies are also being explored to better control the timing of normalization with approaches such as metronomic chemotherapy 164 and nanoconjugates that target the tumor vasculature 165.

### 3.7. Improving TNP delivery via ECM modulation

Alteration of the TME, in particular by altering the makeup of the interstitial ECM, has also been shown to enhance the EPR. For example, angiotensin II receptor blockade using the FDA-approved anti-hypertensive compound losartan can promote vessel perfusion through reduction of stromal collagen and hyaluronan production. Decreased collagen due to losartan treatment can be assessed by SHG imaging 166, 167, which correlated with improved tumor perfusion and PEGylated liposomal doxorubicin (Doxil) uptake 167 (**Figure 7D**). In a subsequent study, Chauhan et al. directly visualized this improved perfusion 166. A recent clinical trial highlights the potential of this TME modulating strategy, demonstrating that combination neoadjuvant FOLFIRINOX and losartan followed by chemoradiation resulted in down staging of locally advanced pancreatic ductal adenocarcinoma and a R0 resection rate of 61% 168. Down regulating the angiotensin II pathway can also modulate the immune makeup of the TME, for example by reducing TAM infiltration 169. Losartan and other drugs with anti-fibrotic effects can reduce interstitial mechanical stress in tumors. Papageorgis et al. modeled this stress alleviation using a similar percolation network model as discussed above 157, solving for blood vessel velocity, IFP and drug delivery parameters 170. This analysis showed that decreased stress resulted in improved tumor vascular perfusion, irrespective of pore size, which is distinct from the process observed for vascular normalization. Improved perfusion, decreased IFP and decreased ECM were simulated and shown to improve tumor penetration of a wide size range of molecules (**Figure 7E**). Correspondingly, experimental data from the same study showed that both nab-paclitaxel (Abraxane, ~10nm) and PEGylated liposomal doxorubicin (Doxil, ~100 nm) had similarly improved efficacy after pre-treatment with tranilast, which is approved for clinical use in Asia and modulates fibrotic and inflammatory responses 170. Cytotoxic chemotherapies such as cyclophosphamide can also alter the tumor IFP to improve NP delivery, likely through pleiotropic mechanisms. Geretti et al. observed this phenomenon using PET with a ^64^Cu-labelled HER-targeted liposomal doxorubicin (MM-302), potentially providing a companion diagnostic to monitor tumor kinetic changes clinically 68. Similar to vascular normalization, a 'priming' window exists for such a strategy: co-administration of cyclophosphamide and MM-302 did not improve TNP uptake, whereas a latency of 2-5 days after cyclophosphamide 'priming' enhanced liposome uptake by 2-3 fold 68. Taken together, these studies highlight the potential of TME modulation for improving NP tumor uptake, and the utility of computational modeling to understand the underlying mechanisms governing such improvements.

### 3.8. Macrophage-mediated dynamic vascular bursts promote TNP delivery

**Figure 8:**
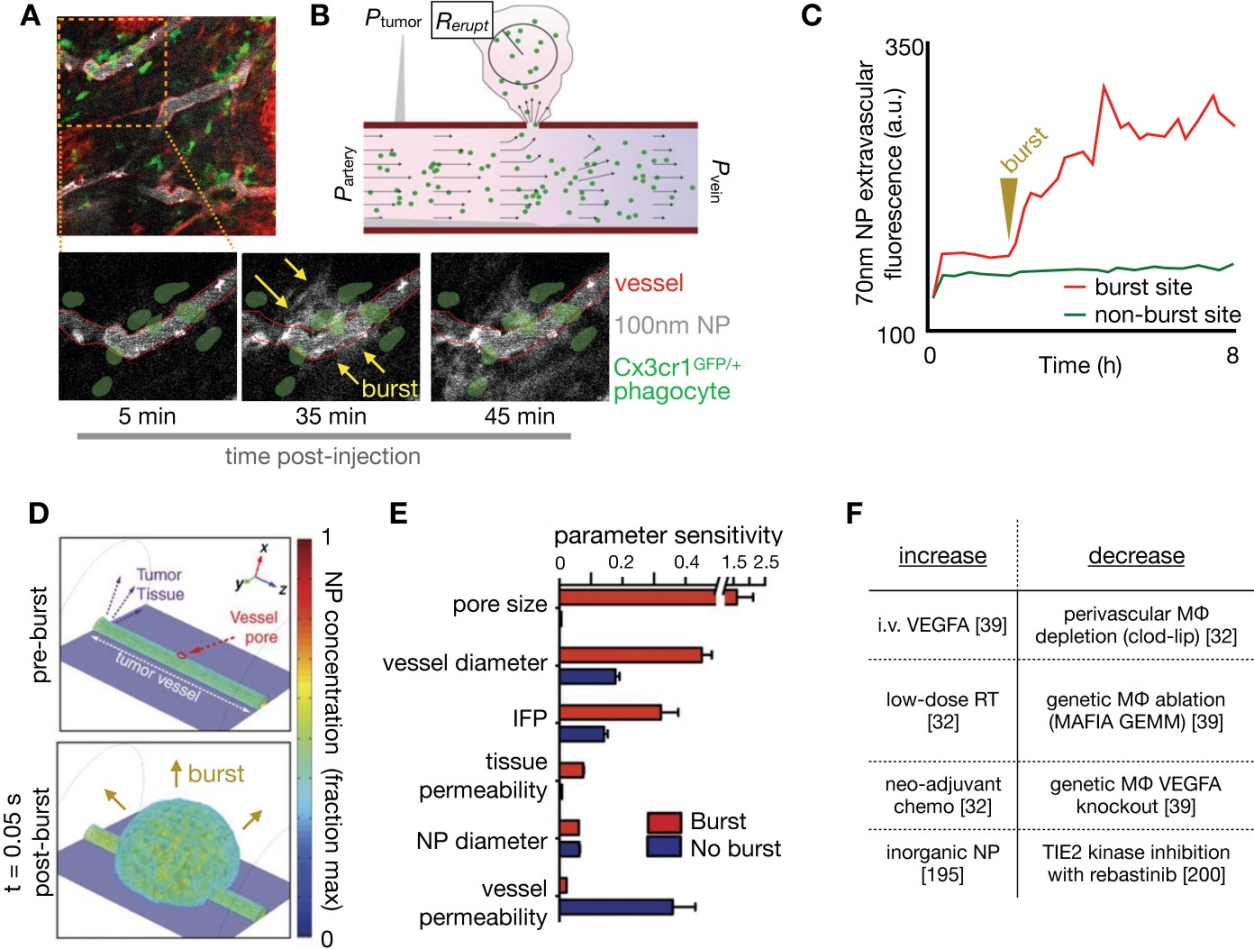
** IVM-guided modeling of vascular bursts as mediators of large NP extravasation.** (**A**) Peritumoral extravasation of ~100 nm polymeric NPs was imaged by IVM, showing a burst of NP extravasation into tissue occurring at a vessel region containing several co-localized GFP+ myeloid cells such as TAMs (Adapted with permission from 32, copyright 2017 AAAS). (**B**) Schematic of a computational model used to simulate vascular bursts. Arterial, venous, and interstitial pressures were fixed to mimic tumor conditions seen from prior studies. Fluid flow (black arrows) outside the vessel is governed by Darcy's law with a fixed, homogenous permeability and driven by pressure gradients. An endothelial gap is opened, NPs (green dots) flow out, and the eruption radius (*R*_erupt_) is quantified (Adapted with permission from 171, copyright 2016 Springer Nature). (**C**) Extravascular NP accumulation was quantified at both eruption and non-eruption sites over time, and such data were used to inform modeling in *B* (Adapted with permission from 171, copyright 2016 Springer Nature). (**D-E**) In a separate study, 3D simulation of vascular bursting activity (D) was used to perform a parametric sensitivity analysis (E), which revealed that pore size, vessel diameter and interstitial fluid pressure were the most sensitive parameters related to burst activity (Adapted with permission from 32, copyright 2017 AAAS). (**F**) In multiple studies, factors have now been reported that increase or decrease vascular bursting.

NP extravasation is not a static nor evenly distributed process. Using IVM, several studies have observed transient and stochastic focal bursts of material extravasation into the tumor interstitial space (**Figure 8A**) 32, 39, 171. Matsumoto et al. modeled these bursts, based upon IVM findings, using a fluid dynamics simulation with particles feeding into flow fields (**Figure 8B**), and noted that this dynamic phenomenon especially affected larger sized NPs (~70nm, **Figure 8C**) 171. The impact of IFP, pore size and tissue porosity upon bursting were examined using a finite element mesh model applied to IVM, with pore size shown to have the biggest impact upon the size and density of bursts. Transcapillary pressure gradients were also found to impact burst density 32, 171. Other studies have examined the molecular and cellular mechanisms underlying vascular burst phenomena. For instance, IVM analysis showed that perivascular TIE2^hi^/VEGFA^hi^ TAMs mediate localized transient blood vessel permeability through VEGFA signaling 39, and radiation therapy (RT) was found to enhance vascular bursting in a manner dependent upon enrichment in perivascular macrophages 32. In a systematic study of high-content IVM data, principal component analysis identified perivascular TAM levels as highly correlative with heterogeneous vascular permeability, which were both among factors most enriched with RT. Based on these results, a 3D nonlinear partial differential equation model of NP transport and extravasation was developed to test various physiological features on vascular bursting behavior (**Figure 8D**). In agreement with Matsumoto et al., this model identified that large (>1 μm) pore size changes were the most sensitive parameter for RT mediated vascular bursting, which occurred on time scales of less than 5 minutes as observed by IVM. Changes in IFP also were predicted to enhance bursting (**Figure 8E, F**), as were larger vessel diameters. Agreeing with the latter, experimental data showed that bursts were in fact more frequently associated with larger vessels, and vessel sizes were on average larger following RT. Of note, model parameter sensitivity analysis in this work found that bursting was relatively less dependent on NP diameter, in large part because the observed burst pores occurred at a μm rather than nm length-scale, and were modeled to indiscriminately allow NPs of various sizes to extravasate.

**Figure 9:**
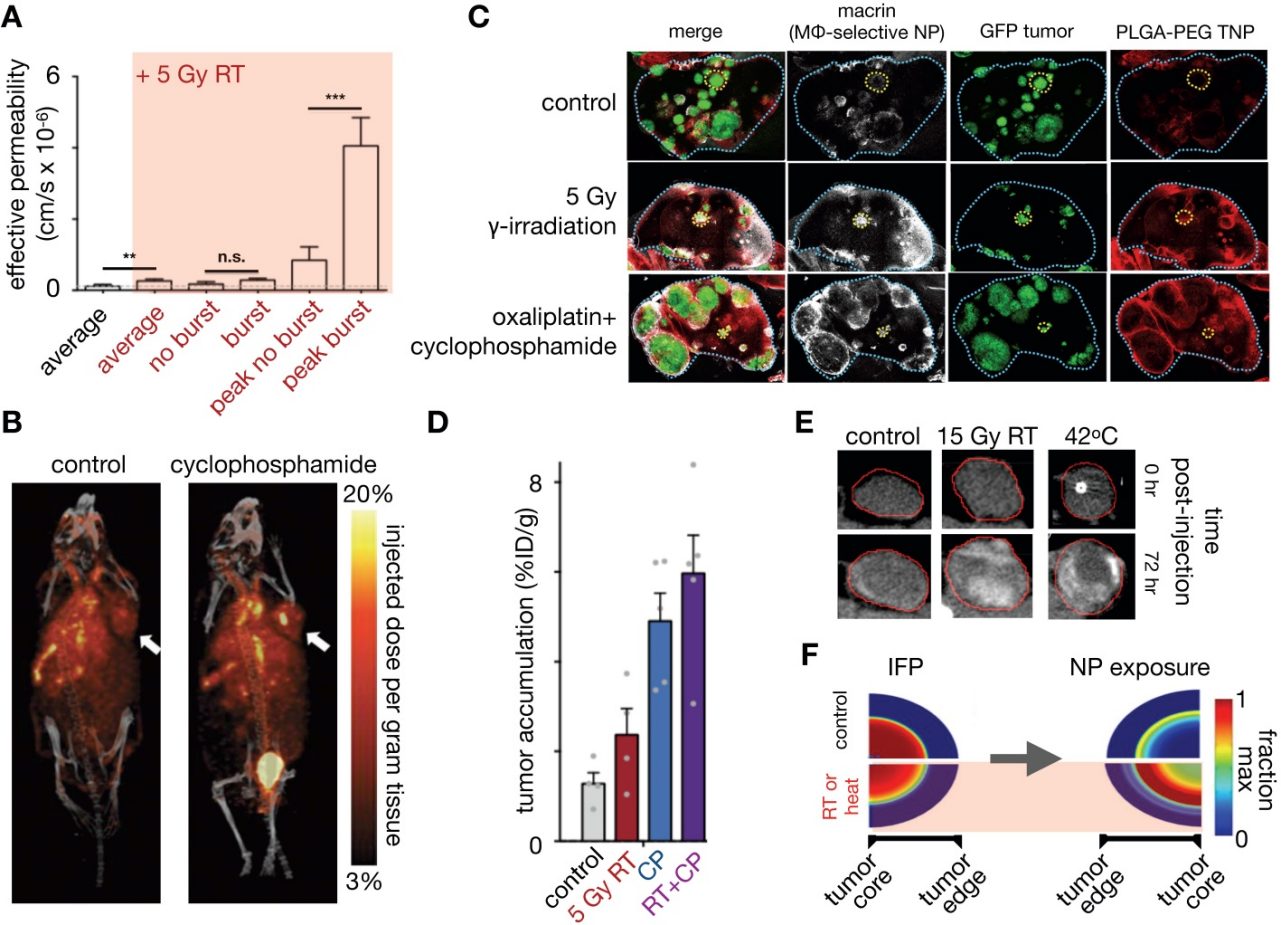
** Tumor priming strategies to modulate EPR for improved NP penetration.** (**A**) A single, relatively low dose of tumor irradiation enhances vascular permeability in a manner that correlates with NP delivery and efficacy. IVM reveals that dynamic vessel bursting leads to peak permeabilities much greater than average levels seen over time (Adapted with permission from 32, copyright 2017 AAAS). (**B**) Cyclophosphamide induces apoptosis, reduced of tumor cell density, decreased IFP, and increased vascular perfusion in tumors. Taken together, these changes improve liposome delivery, as seen here with PET of ^64^Cu-labeled HER2-targeted PEGylated liposomal doxorubicin, one week after cyclophosphamide pre-dosing in a breast cancer model (Adapted with permission from 68, copyright 2015 AACR). (**C**) TAM imaging using the polyglucose NP Macrin correlates with delivery of a model therapeutic NP (based on PLGA-PEG) following radiation or chemotherapy in a model of disseminated lung cancer. Macrin and the PLGA-PEG NP were co-injected 24 hr prior to lung excision, optical tissue clearing, and confocal microscopy. (**D**) Using an FDA-approved liposomal irinotecan formulation (ONIVYDE), maximal tumor uptake was noted when RT and cyclophosphamide (CP) were combined prior to NP administration in a mouse fibrosarcoma xenograft (Adapted with permission from 30, copyright 2018 ACS Publications). (**E-F**) RT and hyperthermia enhance CT-visible liposome uptake in MDA-MB-231 xenografts with high baseline IFP (**E**), and a mathematical model describing fluid pressure, kinetics of NP accumulation, and cell uptake in solid tumors was used to relate imaging to EPR features (**F**). This modeling concluded that elevated IFP results in limited central NP accumulation, but that a transient IFP decrease, such as seen with acute hyperthermia or radiation, can improve the tumor core exposure to NPs (Adapted with permission from 141, copyright 2018 ACS Publications).

Overall, peak bursting activity enhanced by RT resulted in a substantial increase in effective vascular permeability (**Figure 9A**). Experimental results examining the effectiveness of cyclophosphamide and RT in mediating NP uptake corroborated this assertion 30, 32 (**Figure 9B, C),** showing higher TNP uptake and tumor penetration when combinations of neo-adjuvant treatments were used **(Figure 9C, D**). Taken together, these findings implicate several synergistic effects at play with RT treatment that improves NP delivery, combining the effects on NP uptake mediated by vascular manipulation, IFP modulation and direct TNP phagocytosis by TAMs.

The above findings highlight that the functional and anatomic properties of tumor vasculature vary throughout the tumor and over time, even in carefully curated mouse models, let alone among patients. Further, stochastic properties of the vasculature and associated TAM are important for TNP delivery. Thus, developing *in vivo* imaging techniques that identify TAM recruitment 30, quantitatively assess vascular perfusion and permeability, coupled with modeling of realistic vascular structures 46 will continue to play a vital role in refining our understanding of the impact of vasculature in NP delivery.

### 3.9. RT and hyperthermia exert pleiotropic effects on EPR and TNP uptake

Local RT and hyperthermia have been shown to improve TNP uptake in multiple studies 141, 172. In addition to the RT impacts described above, decreased IFP has been posited as a dominant mechanism by which RT and heat can improve tumor TNP delivery: in one report, accumulation of CT-visible liposomes improved towards the center of the tumor with both RT and heat in high IFP tumors, and was associated with a significant decrease in IFP, alterations to tumor vascularity, and improved perfusion (**Figure 9E**)^141^. In contrast, RT and heat did not improve NP penetration in low IFP tumors. To understand these findings, the authors used a previously established biophysical model that described fluid pressure, NP accumulation and cell uptake in solid tumors 173 to simulate the effects of RT and heat. This modeling supported IFP as a dominant factor in impacting NP penetration. More specifically, modeling showed that a rapid transient drop in IFP, as can be mediated by RT and heat, results in improved NP tumor penetration (**Figure 9F**), and spatio-temporal changes in IFP were seen in the animal cohorts in this study. While TAM-dependent effects of RT can build over the course of several days as immune cells accumulate and participate in TME remodeling 30, 32, the more immediate impacts of RT and hyperthermia, for example those that manifest within 24 hours, have shown less reliance on TAM and a greater dependency on IFP and vascular function 141. Given the heterogeneity, complexity, and time-dependence of results, these studies again highlight the need for appropriate companion diagnostics to identify patients/tumors amenable to these tumor-priming strategies. Modeling plays an important role by offering guidance on the type of imaging parameter (e.g. vascularity vs. IFP vs. TAM) that would provide maximal diagnostic information, as well as the timing of such studies.

### 3.10. Amplifying the EPR effect using multistage drug release strategies

**Figure 10:**
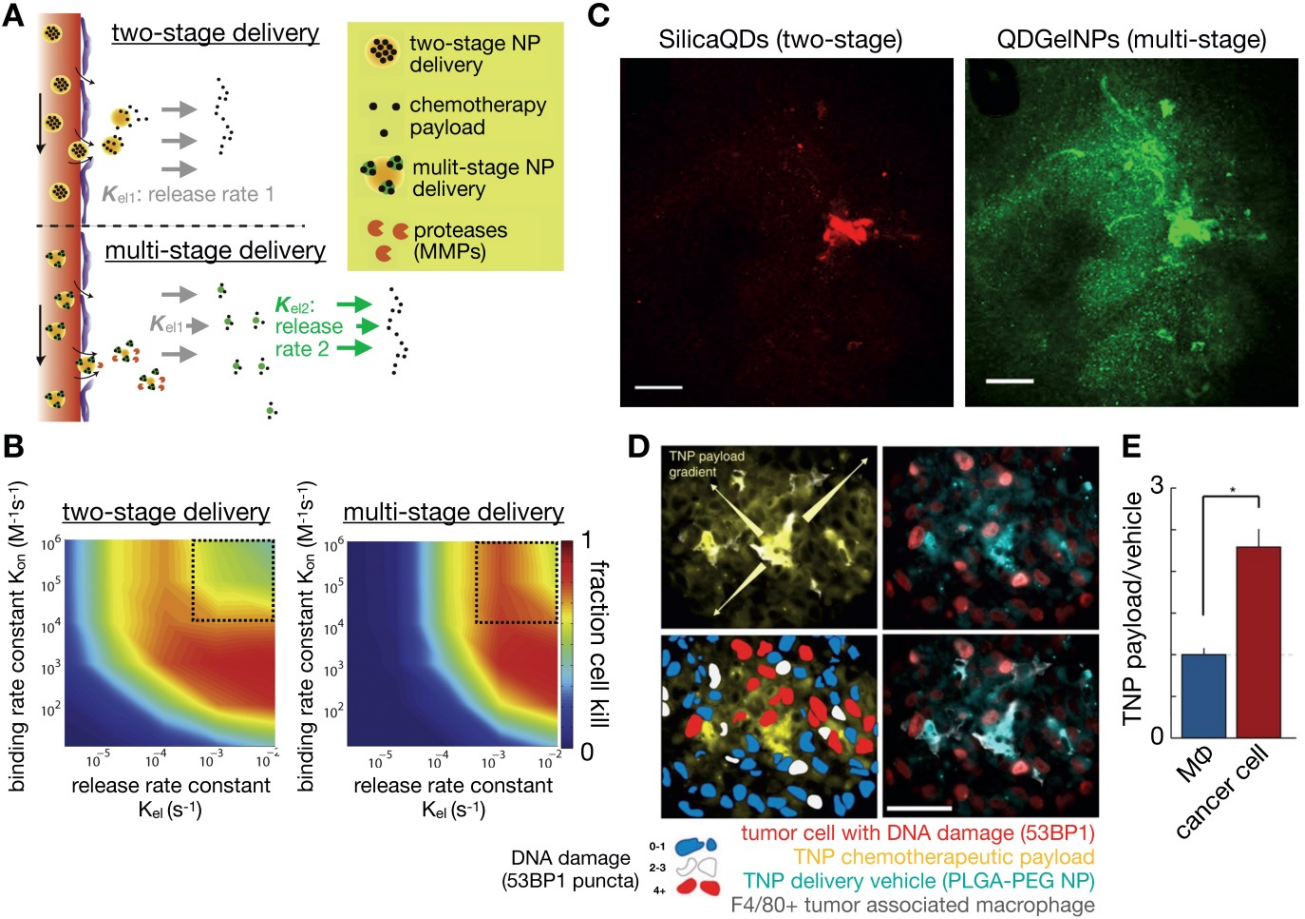
** Image-guided modeling of controlled NP drug release and multistage NP design.** (**A-C**) Conventional two-stage NP delivery systems consist of a nano-carrier and its drug payload (top), while multi-stage systems consist of the primary nano-carrier, a secondary NP, and the drug payload (A). Multi-stage strategies lead to complex changes in how NP parameters relate to overall efficacy, for instance as modeled by cancer cell killing as a function of two parameters (B): NP binding rate constant to target cells, and the drug release rate. Such analysis suggests that the multistage design may be particularly effective under certain parameter combinations (boxed outline), and multistage NP behaviors can be visualized by imaging to quantify improved tumor penetration in a fibrosarcoma xenograft characterized by high MMP2 expression (C; scale bar, 100 μm; Adapted with permission from 177, 178, copyright 2011, 2015 National Academy of Sciences, U.S.A., Springer Nature). (**D-E**) Simultaneous imaging of nano-carriers and their drug payload reveals another example of multistage delivery, whereby NPs are initially taken up by TAMs, which then act as drug reservoirs that release drug payload (here, a DNA-damaging platinum agent) to neighboring cancer cells. (**D**) Polymeric micelle TNP were labeled with two fluorophores, such that the polymer vehicle and cytotoxic cisplatin-related payload could be simultaneously imaged. The DNA damage response marker 53BP1 was used to correlate local drug exposure to response (Scale bar, 50 μm). (**E**) Flow cytometry of excised xenograft tumors showed that cancer cells have more drug payload than TAM, relative to the corresponding amount of NP vehicle in each cell, which combined with imaging (D) indicates that payload redistributed from TAM to tumor cells (Adapted with permission from 31, copyright 2015 Springer Nature).

Novel NP formulations are being designed based upon improved understanding of the EPR effect, and in many examples use environmentally responsive materials that dynamically react with the TME to improve NP penetration and activity. Responsiveness to interstitial acidity and hypoxia 174, metalloproteinases 175, and molecular TME components such as integrins expressed on tumor neovasculature 176 have all been used to physiochemically alter TNP, for instance to enhance payload release, to cause local NP aggregation, or to initiate a multi-step delivery process. Image-guided computational modeling has been useful in understanding the added complexity of these approaches, and in comprehending under what conditions they are most likely to be therapeutically beneficial. One example is the design of a multi-stage NP system 177, comprising large (100 nm) primary NPs that release smaller (10 nm) secondary NPs upon tumor-selective metalloproteinase degradation (**Figure 10A**). In principle, this approach exploits the EPR targeting of the larger sized NP to the bulk tumor mass, while also harnessing the improved diffusivity of the smaller NP within the tumor interstitium (**Figure 10A**). Stylianpoulos et al. computationally modeled the efficacy of such a multi-stage NP design, as compared to a simpler two-stage NP system, using a percolation network model that incorporates fluid flow and NP transport in the tumor vasculature and interstitium, as well as modeling drug release and cellular uptake. This study indicated the multi-stage design to be superior within certain physiologically relevant regimes of NP behavior, and confirmed by experimental IVM studies (**Figure 10B, C**) 178.

Reports have implicated phagocytic TAMs 4, 25, 31 and circulating monocytes 179 as important components affecting tumor NP kinetics, and myeloid NP uptake may likewise be considered one of multiple stages in the delivery of certain TNP designs. Understanding the interaction between TNP and tumor phagocytes is thus vital for understanding pharmacokinetics. For instance, simultaneous IVM examination of both a TNP vehicle and its chemotherapeutic payload, each labeled with different fluorescent tags, revealed preferential uptake of TNPs by TAMs, which then gradually released their drug payload to surrounding tumor cells (**Figure 10D-E**) 31. Downstream tumor cell damage from TNP drug release was also monitored with a fluorescent marker of DNA damage response, which correlated with the spatial gradient of TNP drug payload release 31. Computational modeling has been helpful in quantifying the extent to which phagocytes can play a role in impacting such multistep TNP delivery, and how this role might be predicted in patients. In particular, FMX accumulates highly in TAMs and correlates with TNP delivery in experiments where two NPs have been co-administered. Using IVM-guided distribution of tumor vasculature and TAM, finite element analysis quantified the differences in particular rate constants between FMX and a model TNP based on PLGA-PEG micelles, and these differences among other measurements revealed what fraction of overall TNP delivery is due to indirect TAM-mediated uptake vs. direct TNP uptake by cancer cells themselves 25. Expanding on TAM as an indirect mediator of TNP delivery, myeloid sub-types along a spectrum of polarization phenotypes have been implicated to varying degrees in NP uptake. In particular, alternatively activated, M2-like macrophages have been observed to phagocytose FMX and gold-NP at especially high levels 180, 181. This interaction may be related to the protein corona coating of NPs in the presence of serum 182, may also occur in the MPS, and has been computationally modeled as a function of Fc-receptor mediated uptake 182. Future work should continue to examine how TNP design parameters could be further optimized to leverage their uptake into TAM and such myeloid subsets.

### 3.11. Can phagocytic TNP uptake in the liver vs. tumor be strategically manipulated?

**Figure 11:**
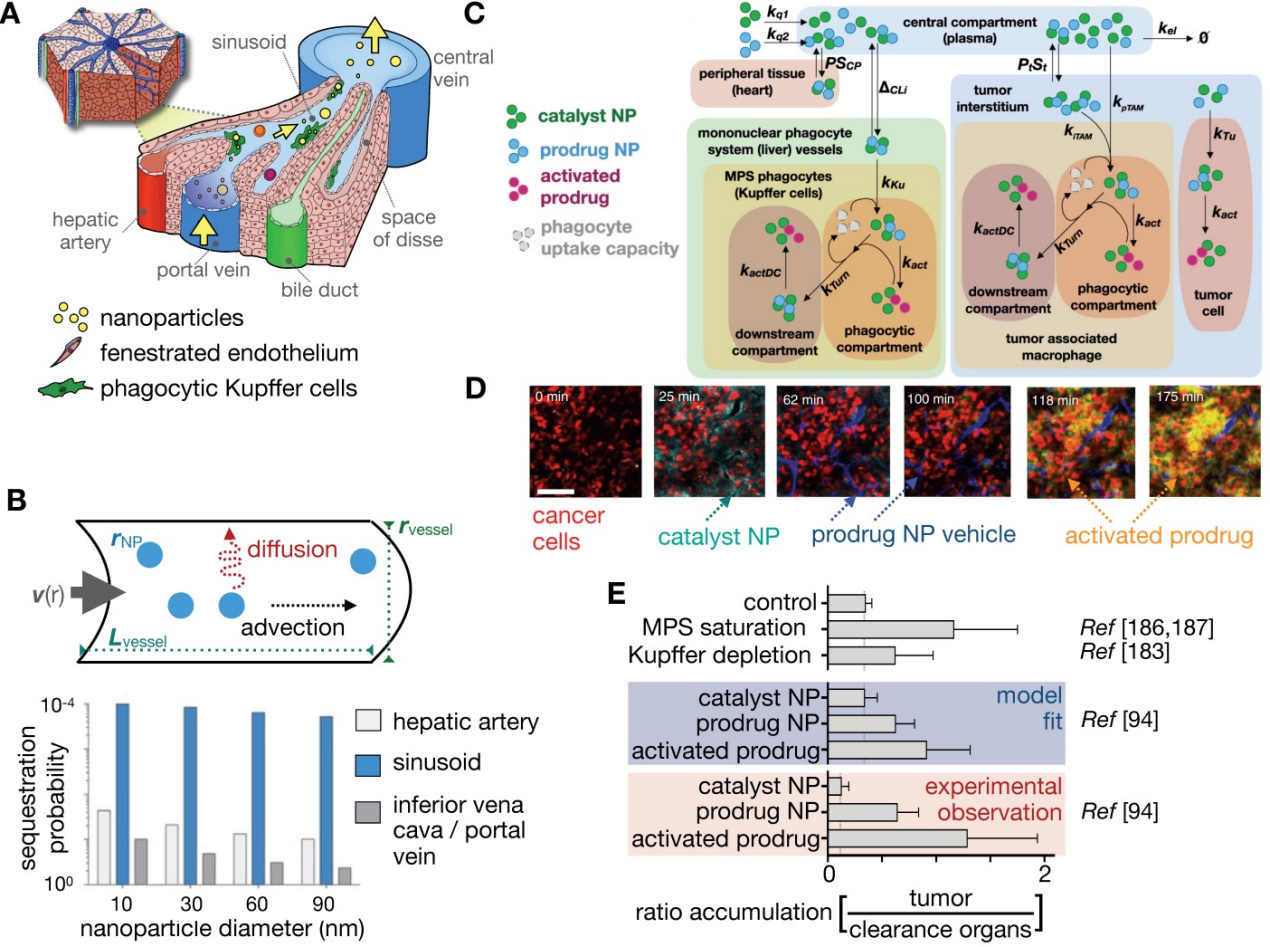
** Understanding and manipulating NP clearance by the mononuclear phagocytic system (MPS).** (**A**) Schematic depicting NP dynamics in the liver parenchyma, with Kupffer cells phagocytosing NPs within the sinusoid. (**B**) Slow sinusoidal blood flow favors diffusive transport, long residence time near phagocytes and corresponding high cellular uptake compared to faster flowing vessels. *In vitro* microfluidics helped support this finding (Adapted with permission from 128, copyright 2016 Springer Nature). (**C-E**) A multi-compartmental model was used to evaluate the balance of systemic NP clearance by the MPS *vs.* NP uptake in TAMs and cancer cells. In particular, the model evaluated how a two-component NP system comprising a prodrug-encapsulated NP, and a second catalyst NP that activated the prodrug, could combine to yield more selective drug activation in the tumor. (**D**) Modeling in *C* was guided by IVM, represented here by time-lapse imaging of an initial catalyst NP, subsequent administration of a prodrug NP in the same subject, and finally followed by activation of the prodrug in extravascular tumor tissue (Adapted with permission from 94, copyright 2018 ACS Publications). (**E**) Comparison of different MPS evasion strategies in enabling selective NP accumulation in the tumor. MPS saturation using pre-treatment liposomes 186, 187, Kupffer cell depletion 183, and prodrug-NP/catalyst-NP administration 94 all showed improved tumor NP delivery compared to controls. In the case of the prodrug strategy, experimental results matched the fit of the computational model in *C*, and further analysis revealed putative mechanisms explaining the enhanced selectivity in delivery.

A significant portion of injected NP dose invariably accumulates in the MPS, predominantly in the liver 6, 128, 183. Image-guided models of liver physiology have been developed for comprehending MPS uptake 128 (**Figure 11A**). In one example, a simple fluid dynamic model linked high MPS uptake to low-velocity vascular flow through liver sinusoids (**Figure 11B**), which was confirmed by *in vitro* microfluidic studies 128. This unique phagocytosis-promoting physiology offers the potential to selectively perturb MPS in the clearance organs rather than in the tumor to improve tumor NP uptake, which is important as TAMs have been reported (as described above) to promote tumoral TNP delivery. Thus while the majority of NP pharmacology studies have focused on the manipulation of the local TME or the NP itself to enhance delivery, reports have also examined MPS manipulation as another means to improve tumor NP uptake (**Figure 11C-E**). Early concepts of MPS manipulation for improved TNP delivery date back to the 1980's, but concerns over systemic toxicity combined with a focus on optimizing NPs themselves largely kept these concepts from advancing 111. While studies have focused on altering the size, charge or the surface functionality of TNPs to avoid MPS clearance 184, these processes can attenuate their desired properties and limit effective tumor targeting. More recently, systemic delivery of lipids via liposomes 185 or intralipid 186 has been proposed as means to saturate MPS phagocytic activity and limit MPS clearance of NPs intended for tumor delivery. In particular, Sun et al. examined the effect of liposome surface charge on their effectiveness at blocking MPS uptake of gold nanorods 187. ^64^Cu-labelled liposomes of different charges clearly showed that positively charged liposomes accumulated more in the liver and spleen, at the expected location of a large number of phagocytes. Coupled with systemic assessment of ^64^Cu-labelled gold nanorod biodistribution with PET, the kinetics and dose of this MPS blockade strategy were assessed with *in vivo* imaging, showing demonstrable improvement of gold NP tumor accumulation. In a similar vein, depletion of liver-resident macrophages (Kupffer cells) also improved tumor to liver NP uptake ratio 183 in a preclinical model; however, systemic depletion of macrophages may face toxicity challenges in patients. NP phagocytosis within the MPS is not limited to Kupffer cells, and other contributing cell populations include liver sinusoidal endothelial cells (LSECs) and hepatic stellate cells 188. The scavenger-receptor *STAB2* acts as the mediator of NP uptake in the zebrafish analog of LSECs, which can be attenuated with dextran sulfate 189. Combined with MPS blockade, this potentially offers a molecular-targeted synergistic strategy to further minimize off-target NP uptake in the MPS.

Building on these studies, computational modeling has been used to gain insights about the efficacy of a dual-NP prodrug/catalyst system and its ability to evade MPS clearance 94. An IVM-guided multi compartmental model that incorporated systemic NP delivery, bulk tumor NP uptake, TAM and tumor cell NP uptake as well as the activation of the prodrug in all compartments (**Figure 11C, D**) confirmed that a dual prodrug-NP/catalyst-NP administration strategy could result in more selective prodrug activation within the tumor, as observed using confocal microscopy and IVM. The computational model reflected experimental results, indicated a reliance on saturating MPS clearance, and thus showed comparable tumor to clearance organ uptake ratio achievable with MPS blockade approaches discussed above (**Figure 11E)**. Building on these imaging results, the model was able to predict an enhanced effect of local RT on tumor activated prodrug uptake and enhanced therapeutic efficacy 94, highlighting the power of image-guided modeling to validate novel NP therapeutic strategies.

## 4. Image guided systems pharmacology approaches for clinical NP translation

Several imaging studies have been performed to understand systemic EPR heterogeneity in patients. Evidence for intra-patient tumor heterogeneity was observed in early studies with ^111^In-labeled liposomes 3. Uptake of radio-labeled PEGylated liposomes also showed high heterogeneity amongst different tumor types, with head and neck cancers showing marked tumor uptake of liposomes compared to breast cancer lesions, even after accounting for tumor size 190. This was in contrast with the relatively uniform uptake in major organs (liver, spleen, lungs, kidneys). Based on these findings, the concept of companion NP imaging diagnostics is being pursued to identify patients who may maximally benefit from TNPs. Giovinazzo et al. used a surrogate agent, ^99m^Tc-sulfur colloid to inform the biodistribution of PEGylated liposomal doxorubicin in ovarian cancer 71. Kinetic analysis of sulfur colloid distribution in the blood, liver and spleen were performed using SPECT/CT and blood sampling, demonstrating a linear relationship between sulfur colloid and PEGylated liposomal doxorubicin clearance in the patients examined. Interestingly, SPECT imaging of patients' hands was related to estimated TNP delivery to the hands and corresponding risk of palmar-plantar erythrodysestesia toxicity. Thus, this study highlighted the potential of imaging to guide TNP dosing based on predicted tumor uptake and off-target toxicity.

**Figure 12:**
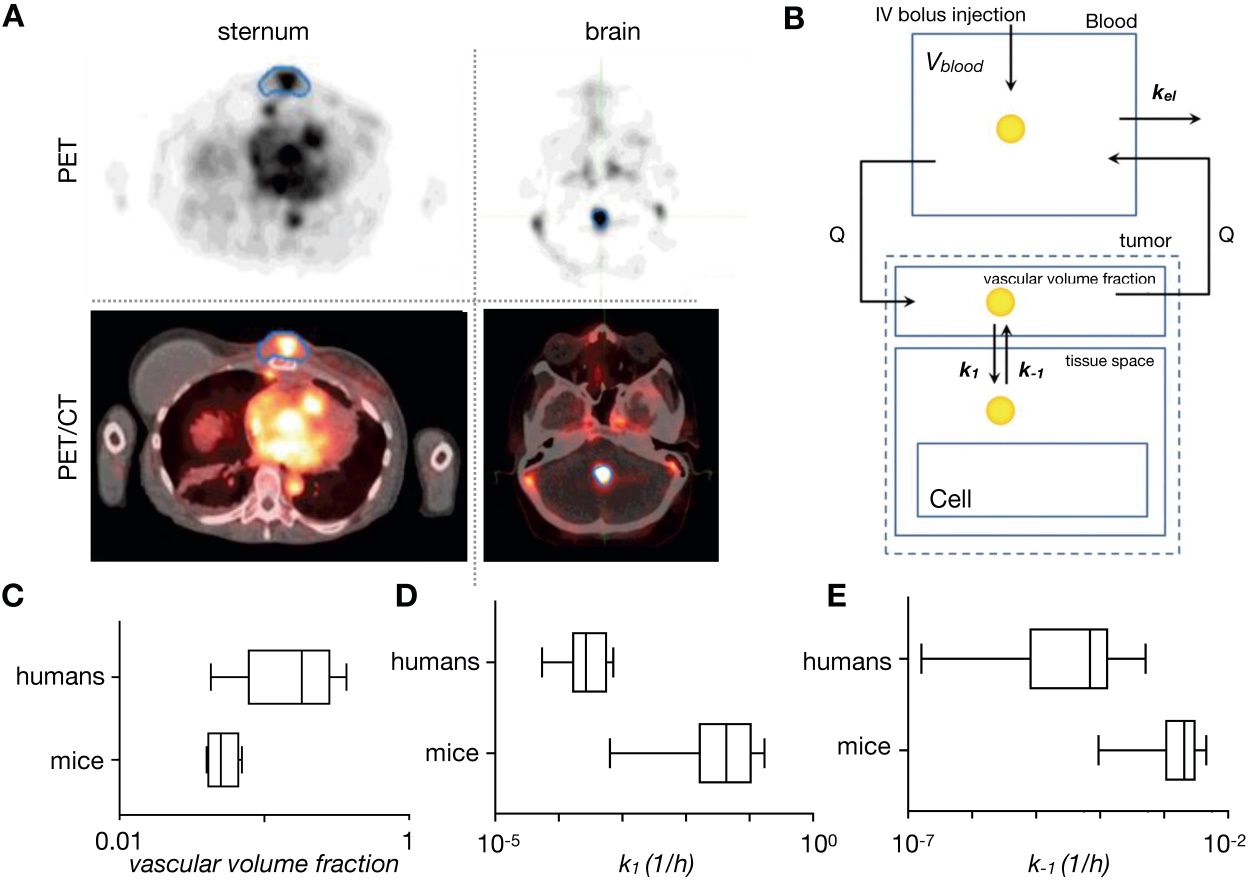
** PET enables quantitative translation of NP pharmacology from preclinical models to patients.** ^64^Cu-MM-302 57, 60 (^64^Cu-labeled HER2-targeted PEGylated liposomal doxorubicin) was developed as a companion diagnostic to understand the distribution of MM-302 *in vivo*, and a drug-free version, MM-DX-929, has also since been developed 69. (**A**) Example PET images from sternal and brain metastases (outlined in blue) show the tumor targeting ability of MM-302. (**B**) A multicompartmental model used to understand the tumor kinetics of ^64^Cu-MM-302 was applied to both clinical and murine preclinical studies (Adapted with permission from 57 copyright 2017AACR). Vascular volume fraction (VVF, **C**), extravasation k_1_ (**D**) and transport out of tumor k_-1_ (**E**) were derived from PET imaging and compared across mice 60 and human tumors 57.

More recently, the MM-302 formulation of PEGylated liposomal doxorubicin, which is targeted against the human epidermal growth factor receptor 2 (HER2), was assessed in clinical trials for advanced breast cancer 191. Lee et al. examined the ability of a ^64^Cu-labelled MM-302 to predict therapeutic efficacy using PET 57, and a companion ^64^Cu-liposome without encapsulated drug, MM-DX-929, has also been tested as a companion imaging agent 69. Significant background uptake of ^64^Cu-MM-302 was noted in the liver and spleen, consistent with preclinical studies 57, 60, and high variability in lesion accumulation of the agent was observed within different tumor locations and between patients (**Figure 12A**). A subset of patients underwent multi-timepoint imaging, enabling compartmental model kinetic analysis that assumed first order clearance from the blood pool and fixed rate of convective transport from the blood pool into the tumor vascular space (**Figure 12B**). This analysis was performed in preclinical studies using the same agent 60, allowing cross-species comparison of kinetic parameters (**Figure 12C**). While the parameters derived from human lesions were more heterogeneous and showed different absolute values compared what was observed in preclinical studies, both analyses highlighted lesion heterogeneity of tumor probe uptake and the utility of imaging at later time points to quantify liposomal tumor accumulation. Similar studies that enable comparison of kinetic analysis between clinical and preclinical studies will allow better insights into the differences in tumor physiology between organisms, complement existing allometric techniques 192 and identify of important parameters that should be targeted in preclinical studies for NP optimization.

**Figure 13:**
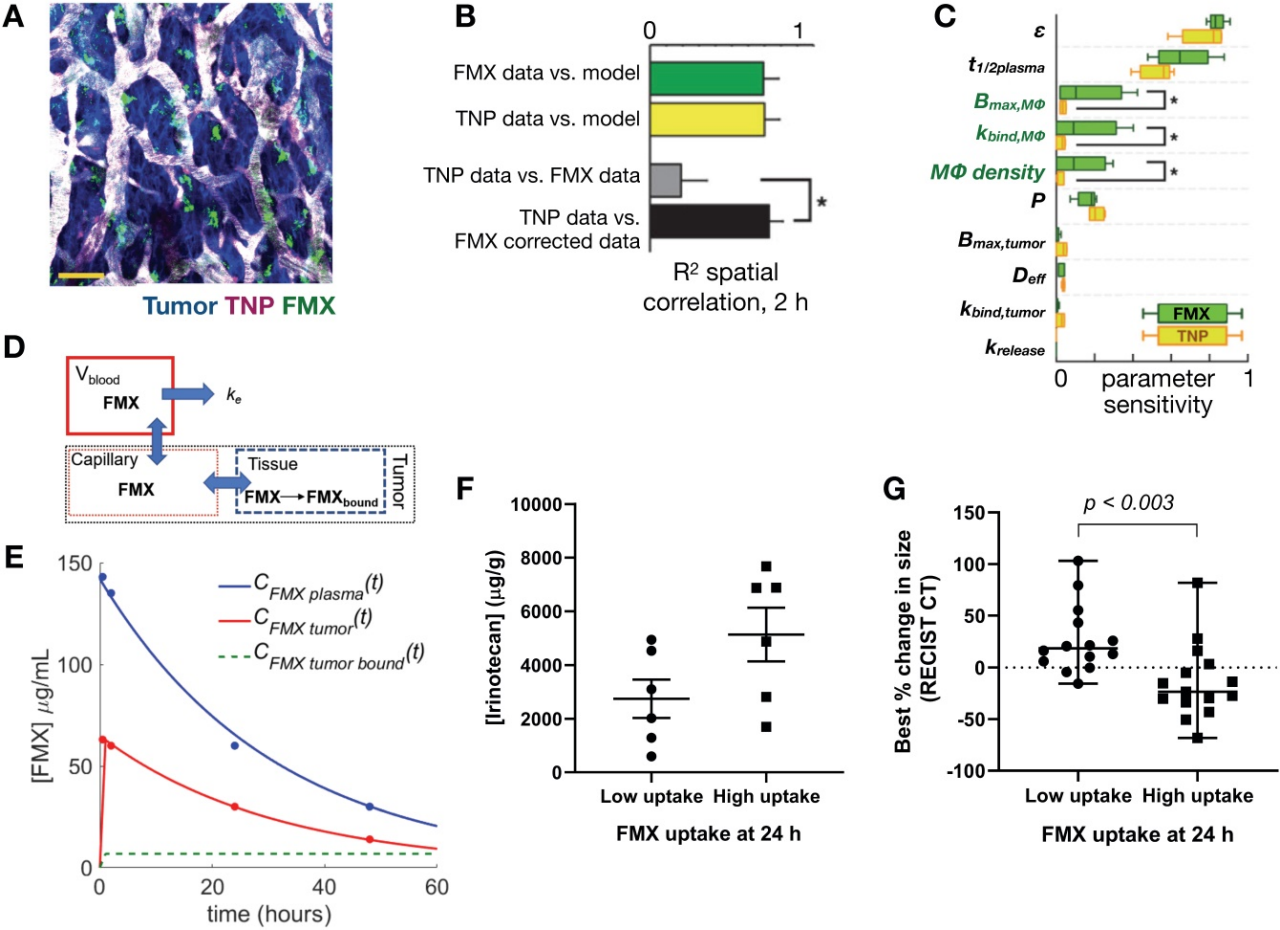
** Ferumoxytol as an MRI companion diagnostic to evaluate NP delivery.** (**A**) IVM simultaneously assessed tumor delivery of fluorescently labeled ferumoxytol (green, FMX) and a model therapeutic NP (TNP, scale bar = 50 μm), which revealed overlap in microscopic cellular uptake patterns of the two NP types. (**B**) Using a finite element model incorporating spatial NP diffusion and heterogeneous NP uptake (guided by IVM), model accuracy in fitting raw imaging data was first assessed (top), and subsequent model fits were used to apply a correction factor that enabled more accurate correlation between FMX and TNP kinetics. Good correlation between FMX and TNPs were observed at spatial resolutions commonly encountered with MRI. (**C**) Model parameter sensitivity analysis at 2 hours post injection showed that extracellular volume fraction in the tissue, ε, and systemic plasma half-life of the NPs, *t*_1/2_ plasma were the most important factors governing tumor uptake. FMX was highly sensitive to macrophage uptake capacity (*B*_max_, MΦ), kinetics (*k*_bind_, MΦ), and density (macrophages per tumor tissue area) at this early time point compared to TNPs (*B*max, maximum NP cellular uptake; *k*_bind_, NP uptake rate; *P*, vessel permeability; Adapted with permission from 25, copyright 2015 AAAS). (**D-E**) A compartmental model to evaluate FMX kinetics proposed by Ramanathan et al 193, with model schematic (k_e_: elimination rate; *D*) and simulated plasma and tumor tissue FMX concentration vs. time curves based on the model equations are shown (*E*). (**F**) In patients receiving liposomal irinotecan therapy, tumor irinotecan levels from biopsy mildly correlated with FMX uptake at 1 (not shown) and 24 h, albeit not in a statistically significant manner. (**G**) Lesions with above median FMX uptake at both 1 h (not shown) and 24 h demonstrated statistically significant best change in lesion size as assessed by RECIST, for patients receiving liposomal irinotecan (Modified from 193).

Imaging agents that efficiently accumulate in TAM have been explored as surrogates to predict NP uptake and subsequent treatment efficacy 25, 30. FMX has been examined using combination MRI and IVM to predict the uptake of TNPs 78. IVM confirmed the colocalization of fluorescently-labeled iron-oxide FMX with model TNPs, especially in tumor associated phagocytes including macrophages. While slight differences in spatial distribution between FMX and their therapeutic counterparts were noted, the two NPs were highly correlated at the spatial resolution of MRI, thereby supporting FMX as a useful companion diagnostic. Clinical studies performed with FMX have also been pursued. Ramanathan et al. explored the use of FMX to predict the uptake and efficacy of nanoliposomal irinotecan 193. While no significant correlation was observed between irinotecan and FMX levels in tumor biopsies, there was a significant inverse correlation between FMX lesion uptake and change in tumor size, thus demonstrating the potential of FMX to predict TNP action. As per previous clinical studies, a wide variability in FMX uptake was noted, emphasizing the heterogeneous EPR effect in patients 193. Computational modeling was applied to the imaging data in the above studies to understand the differences between FMX and TNP kinetics. Finite element analysis modeled the reaction/diffusion parameters for both FMX and TNPs 31, deriving a normalization factor to correct for differences between the kinetics of each type of NP (**Figure 13A, B**). Correction using this factor significantly improved the spatial correlation between FMX and TNP, enhancing FMX's predictive value. The plasma half-life of the agent and extracellular volume within tumor tissue were identified as important EPR parameters governing both NPs (**Figure 13C**). Encouragingly, these findings were consistent with kinetic analysis performed on the clinical data, which showed correlation between liposome-delivered tumor irinotecan levels, change in tumor size, and FMX tumor uptake 193 (**Figure 13D-G**). Subsequent clinical studies showed localization of FMX in TAM 84. Overall, such modeling analyses of the imaging data build on insights into TNP uptake, and allow linkage between the preclinical and clinical studies to be systematically performed.

## 5. Conclusions and future directions

Significant progress has been made in the development of nanotechnologies that target tumors, yet clinical translation of these promising approaches still needs to be improved and accelerated. Successful clinical translation of nanotherapies in the future will need to account for the interactions between the patient and NPs at various levels beyond the local EPR effect alone, including impacts from the mode of NP administration, NP interaction with the MPS, localized delivery to the bulk tumor, and finally internalization into tumor cells and interactions with other components of the TME. As highlighted in this review, imaging tools exist to quantify these interactions at different spatiotemporal scales both preclinically and clinically. Multiple studies to date, many of which have been discussed here, show that the combination of imaging with QSP analysis can and will facilitate the iterative NP development process, yield insights into the physiology of NP delivery, and validate companion diagnostic approaches to predict efficacy.

In the near-term, some key challenges will need to be overcome in order to maximize the potential of an image-guided systems pharmacology approach (**Table 2**). First, as NP kinetic models become more complex, aided by the availability of increased computing power, the amount and quality of robust imaging data required to populate out the models will need to increase. Adoption of novel imaging modalities as well as combining multi-scale imaging approaches (such as MRI with IVM 31) will help address this need. Second, few studies to date link the understanding of NP delivery with NP response, beyond correlation of NP administration with tumor growth delay. Most reports have focused on examining either NP kinetics 72, or pharmacodynamics alone. Continued development of models that link delivery to therapeutic efficacy of the NP-delivered payload will be important to address this issue, particularly at a single-cell level that accounts for intratumoral heterogeneity and immune response. One vital aspect that needs further study is the interaction of NPs with the TME and especially the immune system. For example, NPs carrying drugs designed to modulate TAMs 194 will have a different target objective compared to NPs carrying chemotherapy, and kinetic models used to optimize their delivery should be tailored accordingly. Moreover, TME differences between different tumor models, metastatic and primary lesions, and between model organisms should also be assessed. Applying similar kinetic analyses across different lesions and different species will facilitate this analysis. The impact of NPs on the systems physiology of cancer should also be considered. For example, a recent study implicated the endothelial leakiness induced by titanium dioxide, silica and gold NPs in promoting new metastases in a breast cancer mouse model 195. Finally, incorporating QSP principles in clinical translational studies should be pursued, enabling cross-comparison and standardization between preclinical and clinical studies, as well as between clinical studies across a range of nanomedicines. Taken together, the imaging tools and QSP approaches outlined above should facilitate more effective guidance of promising cancer nanomedicines towards successful clinical adoption and regulatory approval 1.

## Figures and Tables

**Table 1:**
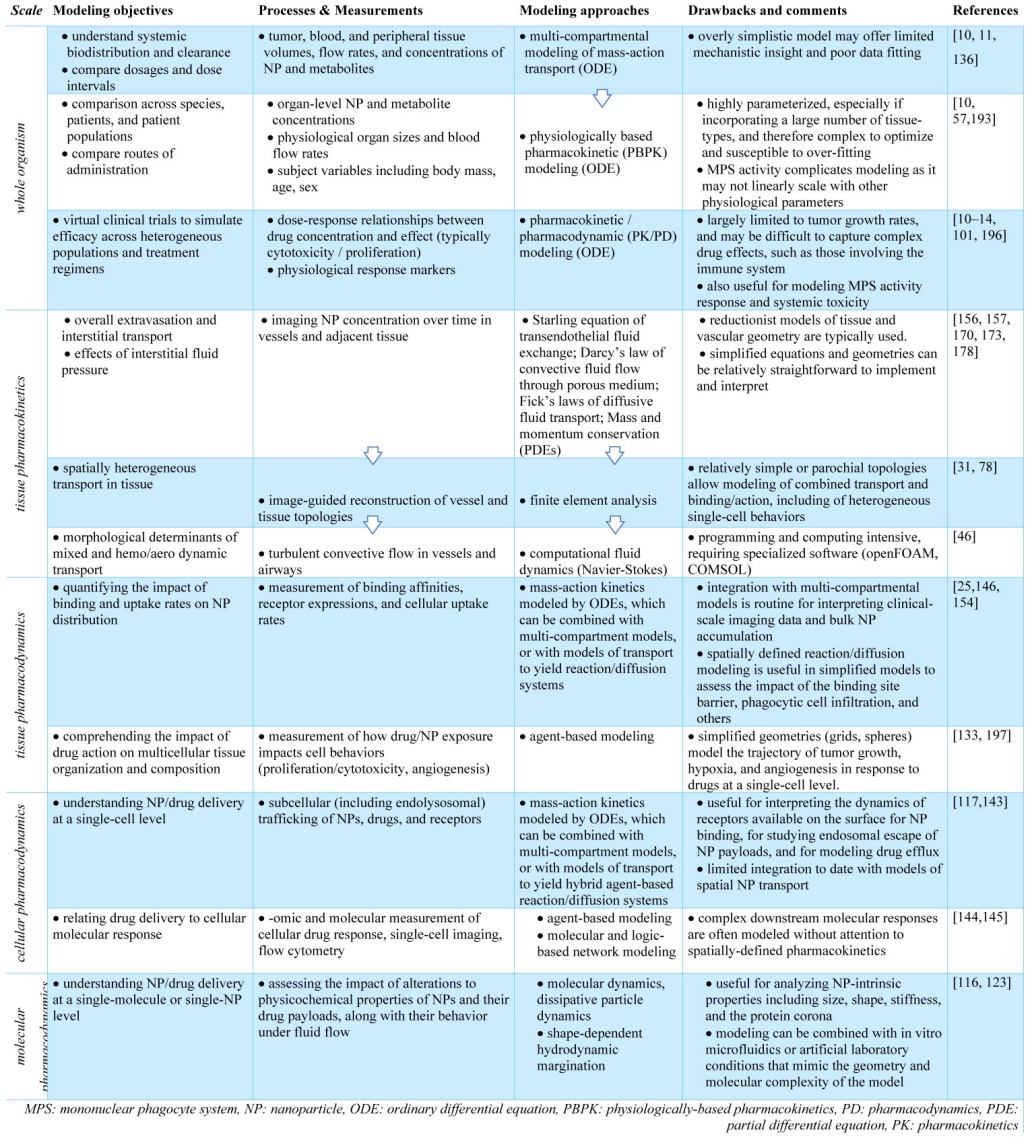
An overview of image-guided computational modeling at multiple scales.

**Table 2:** Challenges and strategies for examining the EPR effect with image-guided systems pharmacology.

Key challenges for imaged-guided systems pharmacology	Approaches to address this challenge
What are limitations of currently existing datasets?	Obtain higher spatial/temporal resolution datasets over large field of views and/or organs Make datasets publicly available
Can imaging technologies provide the necessary information to populate multiscale, physiologically realistic models?	Adoption of novel imaging technologies and adoption of multi-modality approaches to populate model parameters appropriately Build realistic models, harness available computing power
How do NP kinetics in primary and metastatic sites differ?	Orthotopic models, and window chamber IVM Translational imaging with correlative IVM and *ex vivo* assays in patients and animal models
Do findings in preclinical studies (e.g. in mice) reflect what is happening in patients?	Translational imaging with correlative IVM and *ex vivo* assays in patients and animal models Further understanding of allometric scaling and physiological modeling upon NP pharmacology 192 Correlate cellular compositions of mouse and human tumors by multiplexed FNA or biopsies
What are the differences between human and murine tumors?	Translational imaging with correlative IVM and *ex vivo* assays in patients and animal models Comparative analyses (e.g scRNA seq) of TME
Can companion diagnostics identify appropriate patients who will benefit from NPs?	Ensure that the companion diagnostic appropriately reflects the PK of the therapeutic nanoparticle 31 Standardize metrics by which to assess diagnostics performance (likely include modeling parameters, require cooperation of FDA, QIBA, imaging societies)
Does improved delivery (kinetics) equate to response (dynamics)?	Link approaches to image and model drug delivery with those that assess response (especially in the clinical imaging modalities) 4, 25 Include models that assess payload delivery 94
Assess the interaction of NP opsonization with the immune system 130	Super resolution imaging techniques Linking molecular dynamics modelling with higher order models
Assess the effect of nanomaterials on the tumor environment beyond drug delivery 195	Multi-contrast IVM, combined with higher scale imaging modalities (e.g. total body PET) for cell tracking

FDA: Food and Drug Administration, FNA: fine needle aspiration, IVM: intravital microscopy, NP: nanoparticles, PET: positron emission tomography QIBA: Quantitative Imaging Biomarkers Alliance, scRNA: small conditional RNA, TME: tumor microenvironment

## Abbreviations

CT: computed tomography
CLSM: confocal laser scanning microscopy
EM: electron microscopy
EPR: enhanced permeability and retention
FDA: Food and Drug Administration
FDG: fluorodeoxyglucose
FMX: ferumoxytol
GFP: green fluorescent protein
IFP: interstitial fluid pressure
IVM: intravital microscopy
MD: molecular dynamics
MPS: mononuclear phagocyte system
MRI: magnetic resonance imaging
NP: nanoparticle
ODE: ordinary differential equation
PBPK: physiologically based pharmacokinetics
PD: pharmacodynamics
PDE: partial differential equation
PET: positron emission tomography
PK: pharmacokinetics
PLGA: poly(lactic-*co*-glycolic acid)
QSP: quantitative systems pharmacology
SHG: second harmonic generation
TAM: tumor-associated macrophage
THG: third harmonic generation
TME: tumor microenvironment
TNP: therapeutic nanoparticle
VEGF: vascular endothelial growth factor
